# Stable oxidative posttranslational modifications alter the gating properties of RyR1

**DOI:** 10.1085/jgp.202313515

**Published:** 2024-11-05

**Authors:** Maarten M. Steinz, Nicole Beard, Emily Shorter, Johanna T. Lanner

**Affiliations:** 1 Department of Physiology and Pharmacology, Molecular Muscle Physiology and Pathophysiology lab, Karolinska Institutet, Stockholm, Sweden; 2 Faculty or Science and Technology, University of Canberra, Canberra, Australia

## Abstract

The ryanodine receptor type 1 (RyR1) is a Ca^2+^ release channel that regulates skeletal muscle contraction by controlling Ca^2+^ release from the sarcoplasmic reticulum (SR). Posttranslational modifications (PTMs) of RyR1, such as phosphorylation, S-nitrosylation, and carbonylation are known to increase RyR1 open probability (P_o_), contributing to SR Ca^2+^ leak and skeletal muscle dysfunction. PTMs on RyR1 have been linked to muscle dysfunction in diseases like breast cancer, rheumatoid arthritis, Duchenne muscle dystrophy, and aging. While reactive oxygen species (ROS) and oxidative stress induce PTMs, the impact of stable oxidative modifications like 3-nitrotyrosine (3-NT) and malondialdehyde adducts (MDA) on RyR1 gating remains unclear. Mass spectrometry and single-channel recordings were used to study how 3-NT and MDA modify RyR1 and affect Po. Both modifications increased Po in a dose-dependent manner, with mass spectrometry identifying 30 modified residues out of 5035 amino acids per RyR1 monomer. Key modifications were found in domains critical for protein interaction and channel activation, including Y808/3NT in SPRY1, Y1081/3NT and H1254/MDA in SPRY2&3, and Q2107/MDA and Y2128/3NT in JSol, near the binding site of FKBP12. Though these modifications did not directly overlap with FKBP12 binding residues, they promoted FKBP12 dissociation from RyR1. These findings provide detailed insights into how stable oxidative PTMs on RyR1 residues alter channel gating, advancing our understanding of RyR1-mediated Ca^2+^ release in conditions associated with oxidative stress and muscle weakness.

## Teaser

Ca^2+^ release via the ryanodine receptor 1 (RyR1) is essential for skeletal muscle contraction. Here, Steinz et al. combine targeted mass spectrometry and single-channel recordings to elucidate how the stable oxidative posttranslational modifications 3-nitrotyrosine (3-NT) and malondialdehyde adducts (MDA) affect RyR1 channel gating.

## Introduction

The ryanodine receptor type 1 (RyR1) is an ∼2.2 MDa homotetrameric intracellular Ca^2+^ release channel located in the membrane of the sarcoplasmic reticulum (SR) in skeletal muscle (Lanner et al., 2010; des Georges et al., 2016). RyR1-mediated Ca^2+^ release from the SR during excitation–contraction (E–C) coupling enables muscle contraction (Lanner et al., 2010; Gordon et al., 2000). Thus, RyR1 plays a key role in maintaining healthy force production in skeletal muscle. This channel is regulated by a number of factors, including ions (e.g., Ca^2+^ and ATP), co-proteins (e.g., calmodulin [CaM] and the FK506-binding protein [FKBP12]), and posttranslational modifications (PTMs), such as phosphorylation and oxidative modifications (Zanou et al., 2021; Lanner et al., 2010).

Reactive oxygen species (ROS, e.g., ONOO^•−^, O_2_^•−^, NO, OH^•^) are generated by incomplete reduction of oxygen (Cheng et al., 2016). Excessive ROS production and/or reduced endogenous antioxidant defense leads to oxidative stress and can alter and impair protein function (Dalle-Donne et al., 2005). ROS-induced PTMs may induce reversible or irreversible changes in proteins. Reversible changes occur mostly on cysteine residues (e.g., *S*-nitrosylation) and can be reversed by specific enzymes, such as glutaredoxin and thioredoxin (Lillig and Berndt, 2013). Examples of stable ROS-induced PTMs that cannot be efficiently reversed by the antioxidant defense system (Höhn et al., 2013) are carbonylation (DNP) (Lanner et al., 2012), malondialdehyde adducts (MDA) (Place et al., 2015; Cheng et al., 2015), and 3-nitrotyrosine (3-NT) (Yamada et al., 2015; Steinz et al., 2019).

RyR1 is an established redox-sensitive channel, where alterations in oxidative PTMs can result in either channel activation or inactivation (Moore et al., 1999; Sun et al., 2003). For example, sensitized RyR1 activity by *S*-nitrosylation is known to contribute to hyperthermia crises in environmental heat stress and malignant hyperthermia (MH; Durham et al., 2008). Moreover, it is associated with muscle weakness in muscle dystrophy, breast cancer with bone metastases, and normal aging (Bellinger et al., 2009; Andersson et al., 2011; Waning et al., 2015). Increased levels of stable oxidative PTM footprints on RyR1 by DNP, MDA, and 3-NT are also associated with altered SR Ca^2+^ release and muscle function (Lanner et al., 2012; Yamada et al., 2015; Zanou et al., 2021; Waning et al., 2015). However, there is limited knowledge about which specific amino acid residues are modified and their direct effect on RyR1 gating remains elusive.

The small protein FKBP12 (12 kDa) is known to bind RyRs with high affinity, and the stoichiometry of four FKBP12 per RyR1 homotetramer (Ahern et al., 1997a). Removal of FKBP12 from RyR1 leads to greater open probability (P_o_) and longer mean open times of the channel (Brillantes et al., 1994; Bellinger et al., 2008). Markers of oxidative stress on RyR1 are linked to FKBP12 dissociation and altered RyR1-mediated Ca^2+^ release (Waning et al., 2015; Bellinger et al., 2009; Place et al., 2015; Zanou et al., 2021), which also correlate with muscle weakness in breast cancer with bone metastases and ventilator-induced diaphragm dysfunction (Matecki et al., 2016; Waning et al., 2015). However, little is known about how stable 3-NT and MDA modifications affect FKBP12 binding to RyR1 and influence its channel activity.

**Figure S1.**
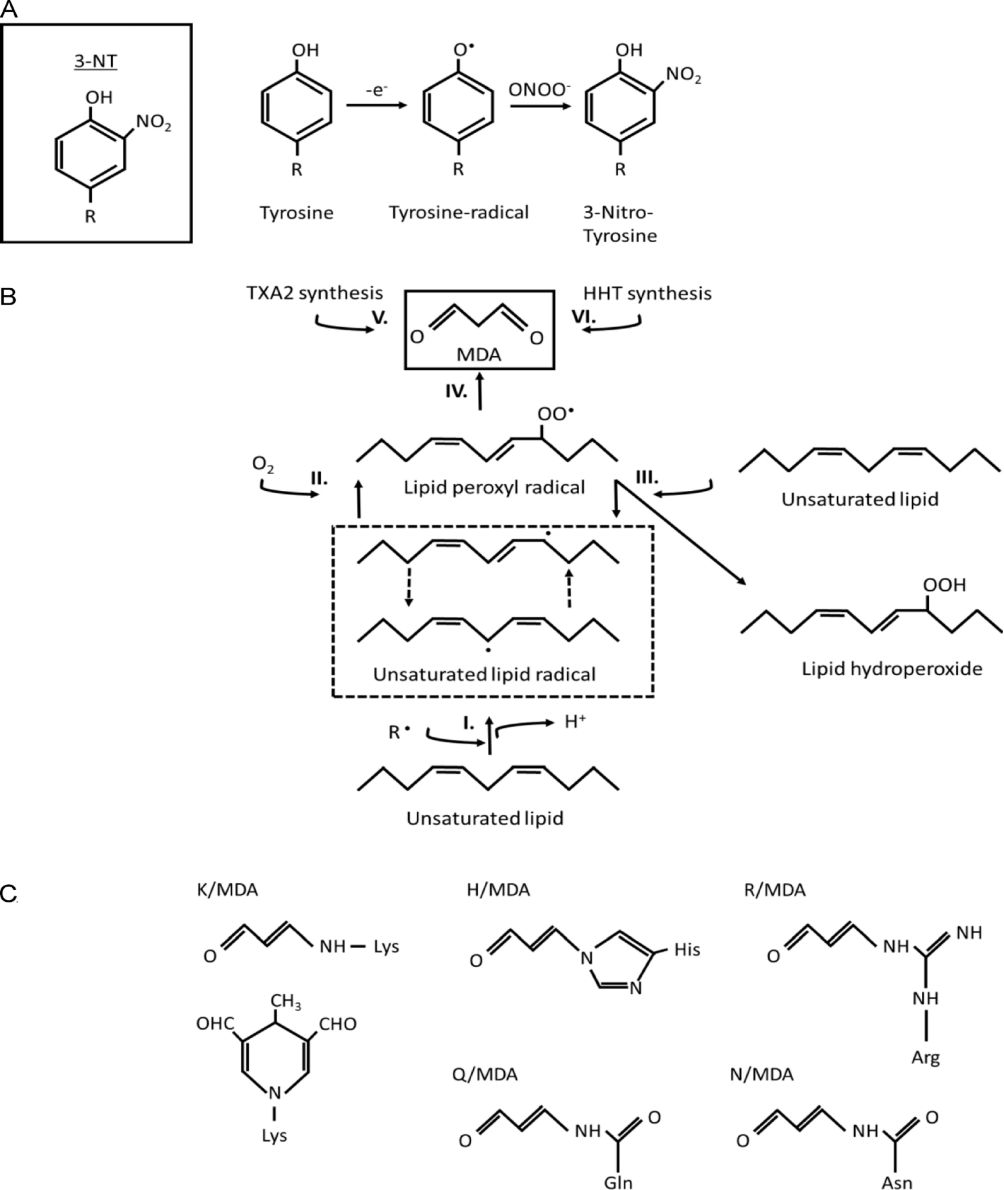
**Simplified chemical reactions for the formation of 3-nitro tyrosine and malondialdehyde.** **(A)** Molecular structure of 3-nitrotyrosine (3-NT). Reactive oxygen species can promote the formation of a Tyrosine-radical (Tyr^•^). Tyr^•^ can react with peroxynitrite (ONOO^−^) which leads to the addition of a nitro-group (NO_2_) at the fifth position on the phenol ring. **(B and C)** Enzymatic and non-enzymatic MDA formation and MDA adducts on amino acids K, H, R Q and N. **(B)** MDA can be formed non-enzymatically through lipid peroxidation. This begins with initiation (I), where pro-oxydants can remove an allylic hydrogen, creating a carbon-centered lipid radical. This radical is then stabilized through a molecular rearrangement, forming a conjugated diene. During the propagation (II) phase, the lipid radical reacts with oxygen, yielding a lipid peroxy radical. This radical then seizes a hydrogen from another unsaturated lipid, producing both a new lipid radical and a lipid hydroperoxide (III). The lipid peroxyl radical can through (IV) cyclic rearrangement, lead to formation of bicycle endoperoxides and subsequent cleavage form MDA. MDA can also be formed enzymatically during the synthesis of (V) thromboxane A2 (TXA2) and (VI) d 12-l-hydroxy-5,8,10-heptadecatrienoic acid (HHT). **(C)** MDA is stable and membrane permeable and can form adducts on basic amino acids lysine (K), histidine (H), arginine (R) and also react with glutamine (Q) and asparagine (N). (Ayala et al., 2014; Zhao et al., 2012; Campolo et al., 2020).

Peroxynitrite (ONOO^•−^) is produced in the presence of NO and O_2_^•−^ (Szabó et al., 2007) and forms 3-NT by nitrating tyrosine (Y) residues (Radi, 2013; Campolo et al., 2020). MDA can form adducts on basic amino acids, such as histidine (H), and can be produced through non-enzymatic peroxidation of lipids or as a byproduct of enzymatic processes such as in the synthesis of thromboxane A2 (Ayala et al., 2014) (see Fig. S1 for simplified chemical reactions). In the present study, we used the NO and O_2_^•−^ donor 5-amino-3-(4-morpholinyl)-1,2,3-oxadiazolium chloride (SIN-1) to generate ONOO^•−^ (Hogg et al., 1992; Martin-Romero et al., 2004) and induce stable oxidative PTMs on RyR1. Targeted mass spectrometry (MS) and single-channel recordings were used to investigate the effects of stable oxidative PTM on RyR1 activity in skeletal muscle. The MS analyses identified 30 modified residues of 5035 amino acids in one RyR1 monomer. These were MDA and 3-NT modifications that were clustered to residues in domains in the cytosolic shell and core region of RyR1, which are known to be important to RyR1 gating. This specific set of oxidative PTMs led to an approximately twofold increase in P_o_ of RyR1. Furthermore, 3-NT and MDA modifications were identified in the cleft that binds the RyR1 stabilizing protein FKBP12, which resulted in FKPB12 dissociation from the channel. The identified oxidative 3-NT and MDA modifications on RyR1 and their effect on channel activity give insight into how stable oxidative PTMs affect the channel open probably and hence promote SR Ca^2+^ leak. This provides key information in the continued efforts to understand the underlying mechanisms of muscle dysfunction in diseases where oxidative stress and altered Ca^2+^ release are part of the pathophysiology.

## Materials and methods

### Animals

All animal experiments were performed in accordance with the Swedish Animal Welfare Act, the Swedish Welfare Ordinance, and applicable regulations and recommendations from Swedish authorities. The Stockholm North Ethical Committee on Animal Experiments approved the use of animals for this study, permit N19/15. The use of animal tissue was also approved by the University of Canberra Animal Ethics Committee (A2012/01). The experiments were performed to meet the guidelines of Replacement, Refinement, and Reduction. The mice were housed in cages with standard rodent chow and water ad libitum with a 12 h light–dark cycle at the animal facility of Karolinska Institutet. The mice were sacrificed by cervical dislocation for the collection of skeletal muscle.

### Preparation of crude SR for mass spectrometry

Crude SR was obtained from pooling eight gastrocnemius muscles from C57BL/6JRj control mice. Snap-frozen muscles were crushed on dry ice with a mortar prior to homogenization. A rotary homogenizer and, subsequently, a Dounce glass homogenizer were used to homogenize the muscles in 20 wt/vol% buffer A containing: (mM) 300 sucrose, 0.5 EGTA, and 20 Tris-maleate (pH 7.4). Then, the homogenate was cleared by 15 min centrifugation at 10,000 × *g* (4°C) and filtered through a cheesecloth. After nutation of the filtrate with KCl (0.5 M, 1 h at 4°C), the samples were centrifuged (Beckman Optima MAX-XP ultra-centrifuge) at 130,000 × *g* for 45 min (4°C). The pellet was washed in buffer B containing 30% wt/wt sucrose and 10 mM MOPS (pH 7.4) and centrifuged again for 45 min at 130,000 × *g* (4°C). The newly formed pellet, containing RyR1 enriched crude SR, was dissolved in the final buffer C containing 300 mM sucrose, 0.1 M KCl, and 10 mM MOPS. All buffers (A, B, and C) contained phosphatase inhibitors (mM), 2 Na_3_VO_4_, 5 NaF, 1 β-glycerophosphate, and protease inhibitor (1 tablet/50 ml; Roche).

### Preparation of enriched SR membrane for single RyR1 channel recordings

Pooled gastrocnemius muscles from four C57BL/6JRj mice were homogenized in a homogenization buffer containing (mM) 300 sucrose and 5 imidazole (pH 7.4), centrifuged for 20 min at 12,000 × *g* (4°C), and filtered. After homogenization of the pellet with homogenization buffer (equivolume to that used in the initial homogenization), the samples were again centrifuged for 20 min at 12,000 × *g* and the pellet discarded. The resultant supernatant was centrifuged (Beckman Optima MAX-XP ultra-centrifuge; TLA100.3 rotor) for 2 h at 43,000 × *g*. The newly formed pellet, containing the RyR1 enriched crude SR, was resuspended in a homogenization buffer (equivolume to that used in the initial homogenization) with a Dounce hand-held homogenizer. All buffers contained phosphatase inhibitors (mM), 2 Na_3_VO_4_; 5 NaF; and protease inhibitor cocktail (containing 1 µM leupeptin, 1 µM pepstatin, 1 mM benzamide, 0.7 mM PMSF).

Rapamycin (or vehicle) treatment of vesicles was conducted in accordance with Ahern et al. (1997b) with minor changes. In brief, vesicles (2 mg/ml) were incubated in homogenization buffer containing either 5 mM rapamycin (from a 5 mg/ml stock where rapamycin was dissolved in ethanol) or 0.94% ethanol (for vehicle-treated) for 1 h at room temperature (RT). Treated vesicles were sedimented by centrifugation at 130,000 × *g* (Beckman 154 Optima MAX-XP ultra-centrifuge; TLA100.3 rotor) for 15 min at 4°C. The resultant pellet was washed once in homogenization buffer and again sedimented by centrifugation to separate the vesicles from dissociated FKBP12. The resultant pellet was suspended in a homogenization buffer at ∼2 mg/ml. Samples were then incubated in dithiothreitol (DTT) and SIN-1 as per section 2.4 for single-channel experiments.

### SDS page and immunoblot

Proteins were separated on SDS 4–12% gradient gels by electrophoresis and transferred to a PVDF membrane prior to immune blotting with the antibodies: 3-NT (Ab52309; Abcam); RyR1 (34C Developmental Studies Hybridoma Bank); and FKBP12 (sc-133067; Santa Cruz Biotechnology, Inc.). The membrane was washed with tris-buffered saline with 0.05% tween and incubated with a secondary IgG HRP conjugated antibody (Thermo Fisher Scientific). Chemiluminescent signals were read with a LI-COR Odyssey Fc Imaging System. The chemiluminescent signal intensity of the immunoreactive bands was analyzed by use of the LI-COR Image Studio Lite software and normalized against an antibody-detected RyR1 (total RyR1). In the case of experiments using 3-NT, blots were stripped and reprobed with anti-RyR1 antibody 34C) to confirm the identity of RyR1 as a nitrated protein.

### Incubation of crude SR in SIN-1

For single-channel experiments, enriched SR membranes were preincubated in 1 mM DTT and subsequently in 1 mM SIN-1 (Cayman chemicals) (1 mM) at RT for 1, 5, 15, or 120 min. Crude SR was incubated with SIN-1 (Cayman chemicals) (10 mM) at RT for 15 min.

For SDS PAGE and immunoblot experiments, SR vesicles were suspended in a MOPS buffer (150 mM NaCl, 20 mM MOPS, pH 7.4) at 2 mg/ml. Enriched SR membranes were preincubated in 1 mM DTT and subsequently in 1 mM SIN-1 at RT for 1, 5, 15, or 120 min. Treated vesicles were washed with MOPS buffer (5× vol) in an Amicon Ultra Centrifugal Filter (at ∼10,000 rpm for 5 min) to remove any dissociated proteins and SIN-1. The retentate was subject to SDS Page and immunoblot.

### Single-channel recordings

Single RyR1 channel recordings were performed in a lipid bilayer that separates a *cis* (cytoplasmic) (230 mM CsMS, 20 mM CsCl, 1 mM CaCl_2_, and 10 mM TES; pH 7.4) and a *trans* (luminal) (30 mM CsMS, 20 mM CsCl, 1 mM CaCl, and 10 mM TES; pH 7.4) solution. The SR membranes (enriched in RyR1 channels) were added to the *cis* solution so that the cytoplasmic surface of RyR1 was positioned toward the *cis* solution after incorporation in the lipid bilayer. *Trans* [Cs^+^] was then raised from 50 to 250 mM with the addition of 200 mM CsMS. In the *cis* solution, 2 mM ATP and ∼1.3 mM 1,2-bis(o-aminophenoxy)ethane-N,N,N′,N′-tetraacetic acid (BAPTA) was added to achieve a final free [Ca^2+^] of 10 μM. The addition of vehicle (PBS), 1 mM DTT, and 0.2, 1, or 10 mM SIN-1 were made to the *cis* chamber where appropriate. The orientation of the incorporated RyR1 was confirmed by the characteristic responses of the channel toward changes in cytoplasmic [Ca^2+^], ATP at the beginning of the experiment, and ruthenium red at the end of experiments. Single-channel recordings were obtained at +40 mV and −40 mV at RT (23 ± 2^ο^C). These voltages ensured a large current flow through the channel, maximizing the signal-to-noise ratio and minimizing the risk of breaking the bilayer. Data were sampled at 5 kHz, filtered at 1 kHz for 90–180 s, and analyzed using the Channel 2 program (developed by P.W. Gage and M. Smith, John Curtin School of Medical Research, Canberra, Australia). Baseline noise was excluded and defined as ∼20% of the maximum single-channel conductance and measured P_o_. When two or more channels were active, P_o_ was approximated from the fractional mean current, I′_F_, which reflects P_o_ and is equal to P_o_ under ideal conditions. All electrical potentials are expressed as that of *cis* solution relative to the *trans* solution.

### Mass spectrometry

MS was performed on RyR1 from crude SR. Samples were prepared as described previously (Steinz et al., 2019). In brief, trypsin-digested peptides were separated by chromatography and electrosprayed in an Orbitrap QExactive Plus mass spectrometer (Thermo Fisher Scientific). The survey MS spectrum was acquired at a resolution of 60,000 (m/z range 200–2,000). RawtoMGF software was used to extract the MS/MS data and Mascot (Matrix Science, version 2.5.1), and X!Tandem (https://www.thegpm.org/; version CYCLONE 2010.12.01.1) software was used for analysis. On average (*n* = 3), we obtained 58 ± 1.5% coverage of RyR1 in the mass-spec analyses. Tyr-nitration (−NO_2_; +46 Da), Cys-nitrosylation (−NO, +30 Da), and MDA (−C_3_H_3_O; +54 Da) were specified as variable PTMs, i.e., modifications that were introduced independent of the sample preparation. Cys-carbamidomethylations were specified as a fixed modification, i.e., modifications introduced by the sample preparation. DNP could not be detected as it requires a targeted approach with labeled reaction sites. MS/MS-based peptides, proteins, and posttranslational modifications were validated with Scaffold (version Scaffold_4.4.5; Proteome Software Inc.). Protein probabilities were assigned with the protein prophet algorithm (Nesvizhskii et al., 2003).

### Chimera 3-D protein modeling

Clustal sequence alignment (Larkin et al., 2007) and the UCSF-Chimera software (Pettersen et al., 2004) were used to find the location of the oxidative 3-NT and MDA modifications within the 3-D structure of rabbit RyR1 PDB-EBI files 7m6a (Melville et al., 2022) and 3J8H (Yan et al., 2015). Modifications are presented with their one-letter code and specifier.

### Statistics

**Figure 1.**
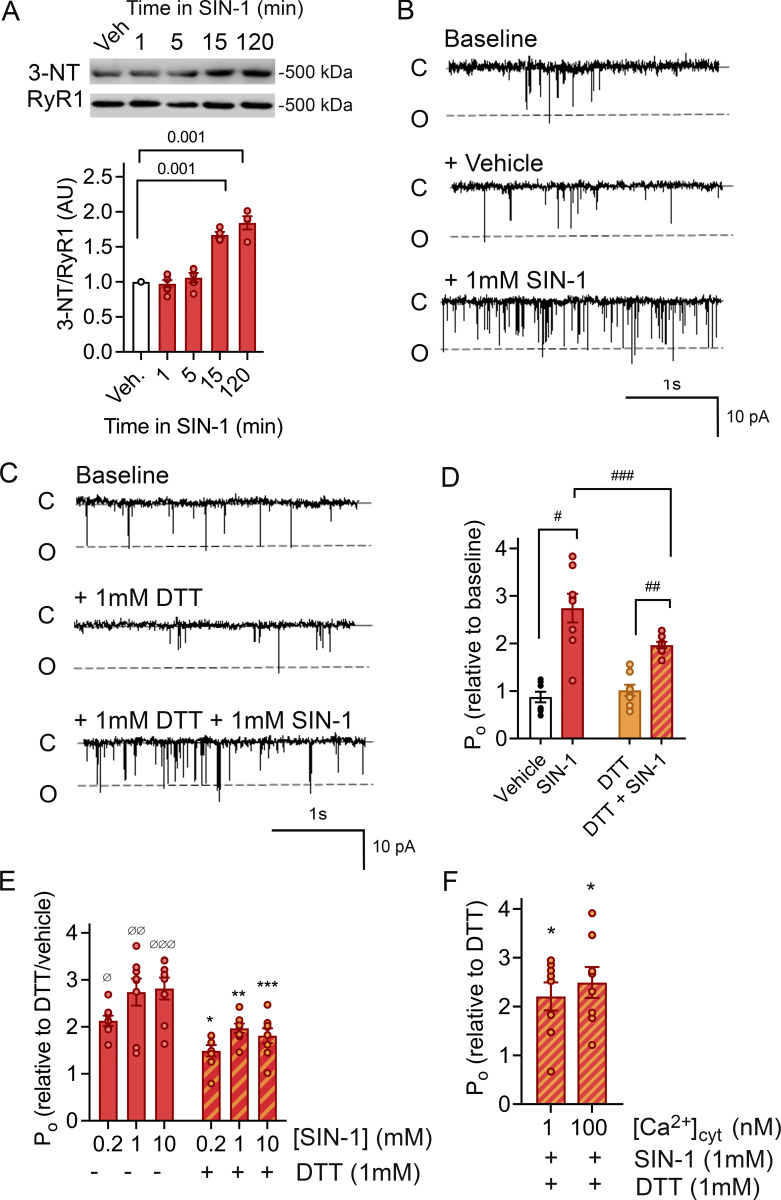
**SIN-1 increases RyR1 open probability. (A)** Immunoblot and mean ± SEM of 3-NT levels of RyR1 incubated with SIN-1 (1 mM). P < 0.001 15 and 120 min, *n* = 4–6 for each condition, across six immunoblots. Approximate molecular weights are to the left of immunoblots. **(B and C)** Typical records of RyR1 channel recordings. Channel opening is downward from zero current (c, solid line) to maximum open conductance (o, dashed line) at −40 mV. Channels were recorded in symmetrical 250 mM CsCH_3_SO_3_, pH 7.3 buffer. *Cis* (cytoplasmic) and *trans* (luminal) [Ca^2+^] are 10 µM and 1 mM, respectively. Baseline activity (top), after addition of vehicle (middle) and subsequent addition of 1 mM SIN-1 (bottom) (B) or after addition of 1 mM DTT (middle) and subsequent addition of 1 mM SIN-1 (bottom) (C). P_o_ of individual channel traces in B are 0.07 (baseline), 0.07 (vehicle), and 0.16 (1 mM SIN-1) and C are 0.05 (baseline), 0.04 (1 mM DTT), and 0.14 (1 mM DTT + 1 mM SIN-1). **(D)** Relative P_o_ (mean ± SEM, *n* = 8) from measurements of P_o_ at +40 and −40 mV showing the open probability (P_o_) measured under conditions shown in B and C and presented as relative to that recorded under baseline conditions. There was no difference in channel responses at +40 or −40 mV. ^#^P = 1.20 × 10^−6^ between vehicle and SIN-1; ^##^P = 2.61 × 10^−11^ between DTT and SIN-1; ^###^P = 2.3 × 10^−4^ between SIN-1 (no DTT) and SIN-1 (DTT). **(E)** Relative P_o_ (mean ± SEM, *n* = 8) was measured in the presence and absence of 0.2, 1.0, and 10 mM SIN-1. P_o_ is presented as relative to that recorded in the presence of vehicle or DTT alone. ^Ø^P = 1.17 × 10^−6^ for 0.2 mM SIN-1, ^ØØ^P = 2.38 × 10^−9^ for 1 mm SIN-1 and ^ØØØ^P = 7.79 × 10^−10^ for 10 mM SIN-1 (no DTT). *P = 0.05 for 0.2 mM SIN-1, **P = 0.002 for 1 mm SIN-1 and ***P = 0.001 for 10 mM SIN-1 (no DTT). **(F)** Relative P_o_ (mean ± SEM, *n* = 8) in the absence and presence of SIN-1 with either 100 or 1 nM Ca^2+^. P_o_ is presented as relative to that recorded in the presence of DTT alone, at the respective [Ca^2+^]. In comparison to vehicle, a significant increase in P_o_ with 1 nM Ca^2+^ (P = 0.03) and 100 nM Ca^2+^ (P = 0.03) was present, but no difference in P_o_ between the low and high Ca^2+^. Source data are available for this figure: SourceData F1.

Average data is presented as mean ± standard error of the mean (SEM). For western blots, significance was evaluated using a one-way analysis of variance (ANOVA) with a Tukey’s honest significant difference post hoc test. For single channels, significance was tested using one-way ANOVA with Bonferroni’s post hoc analysis (Fig. 1, A, D, and E; and Fig. 3 F) or using Student’s *t* test using XLSTAT. P ≤ 0.05 was considered significant.

### Online supplemental material

**Figure S2.**
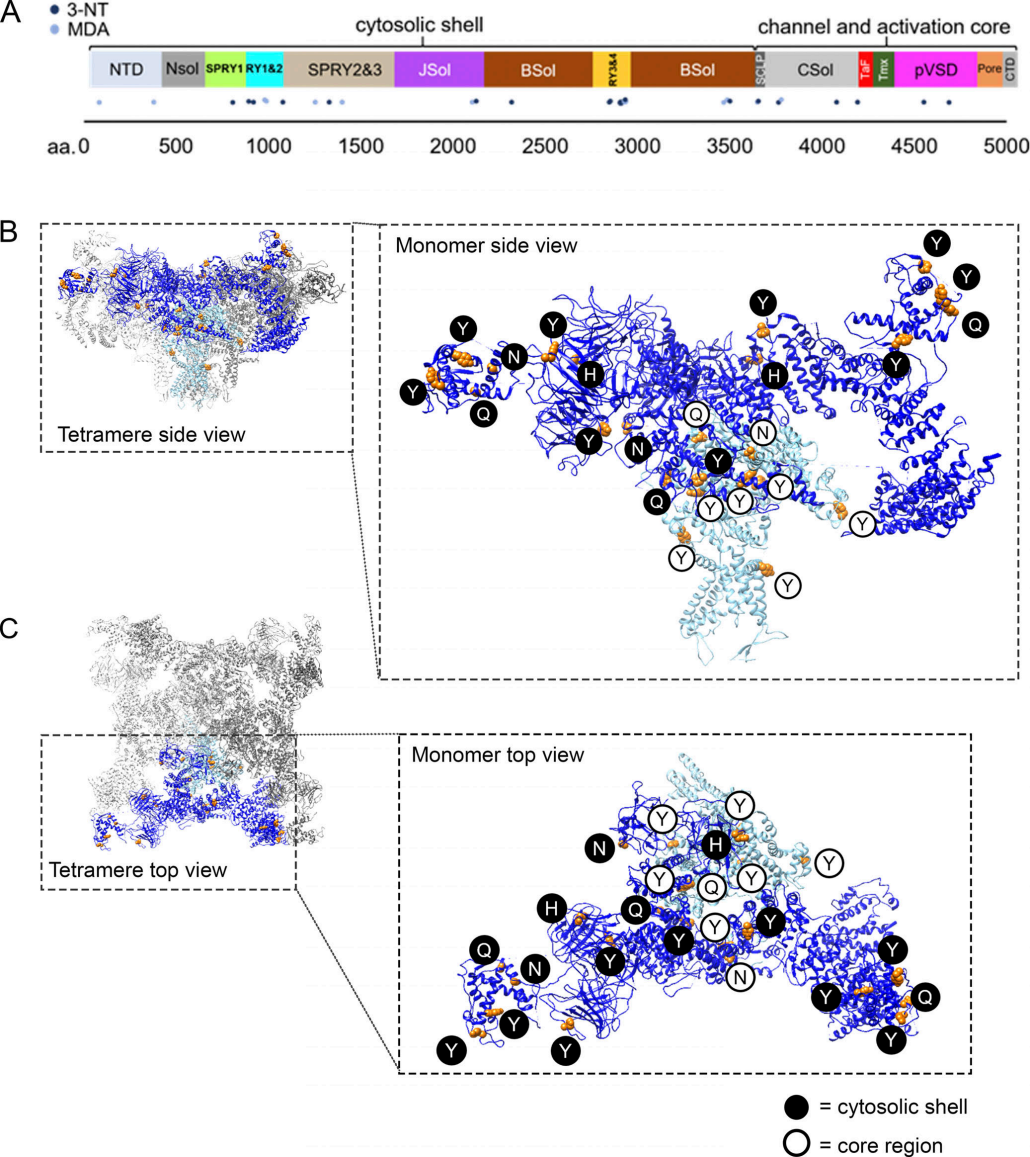
**The position of the identified oxidative 3-NT and MDA PTM on RyR1.** **(A)** Linear overview of the RyR1 sequence with the globular domains highlighted in color. Skeletal muscle crude SR incubated with SIN-1 ([10 mM], 15 min at RT, *n* = 3) were analyzed with mass spectrometry and the identified oxidative PTM on RyR1 are represented by dots over the linear sequence (dark blue: 3-NT, light blue MDA). Of the 30 identified PTMs (see Table S1), 26 (orange) have been structurally determined andcould be mapped in the RyR1 crystal structure adapted from Melville et al. (2022) with UCSF Chimera (Pettersen et al., 2004). **(B and C)** Side (B) and top (C) view of the oxidative PTMs (orange) on one RyR1 monomer. The cytosolic shell is depicted in dark blue and modification in this part are in dark blue circles. The core is depicted in light blue and the same color is used to highlight modifications that were identified in this part of the protein.

**Figure S3.**
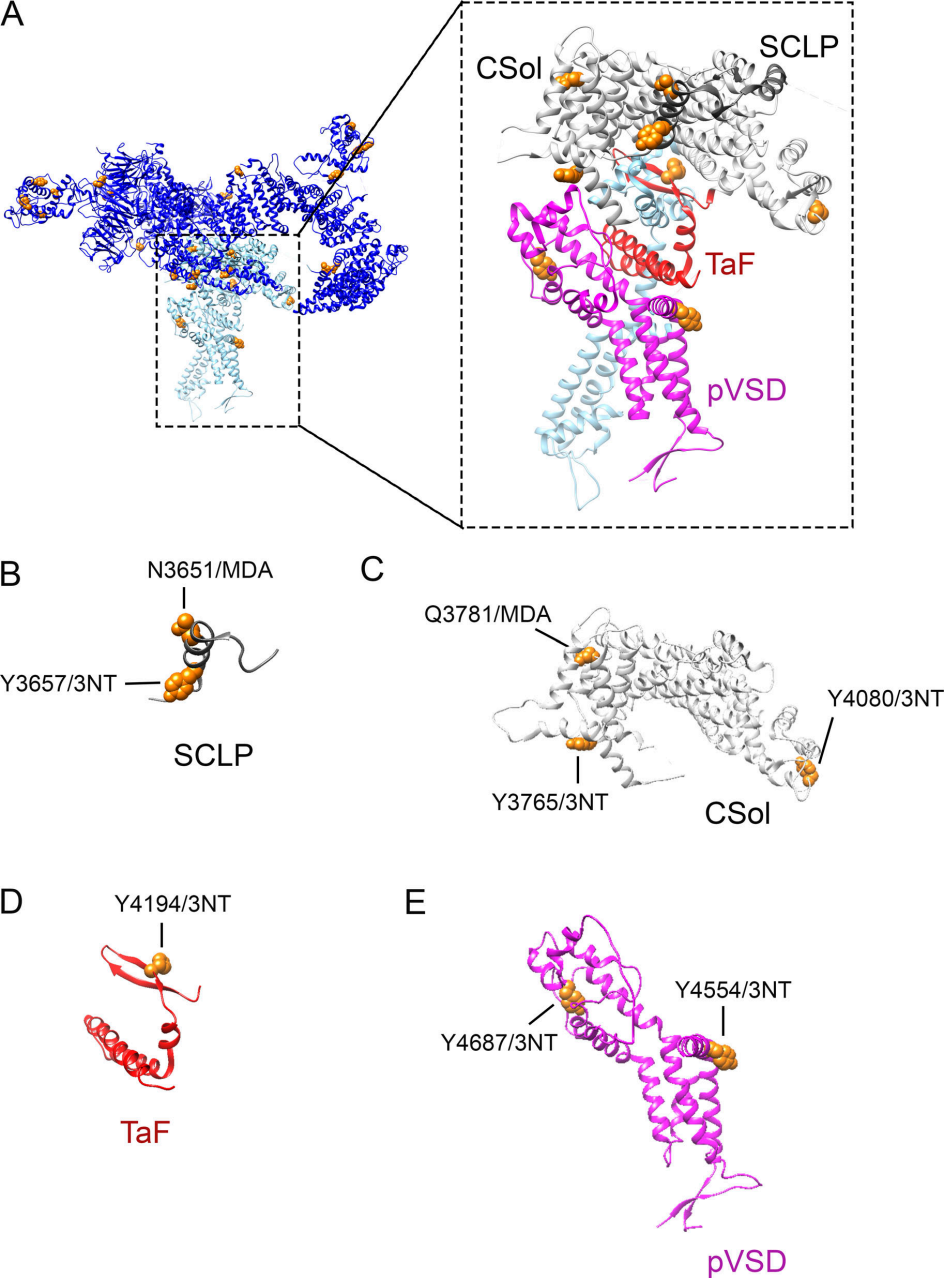
**Oxidative modifications localized to the channel and activation core of RyR1.** **(A)** Overview of the oxidative PTM induced by SIN-1 in the channel and activation core of the RyR1 monomer with colouring of the different domains, which were kept consistent throughout the figure. The RyR1 cryo-EM structure was adapted from the Melville et al. (2022) (PDB-EBI 7m6a) and the location of the modifications was determined by Clustal protein sequence alignment of the mouse and rabbit protein sequence of RyR1. Tyrosine residues (Y) were nitrated (3-NT), whereas and histidine (H), glutamine (Q) and asparagine (N) were MDA modified. **(B–E)** 3-NT and/or MDA modifications recurred in SCLP (B), CSol (C), TaF (D) and pVSD (E). The 3-NT and MDA oxidative modifications were identified by mass spectrometry on RyR1 from mouse skeletal muscle crude SR that had been incubated in SIN-1 ([10 mM], 15 min at RT, *n* = 3). The models were generated with UCSF-Chimera (Pettersen et al., 2004).

**Figure S4.**
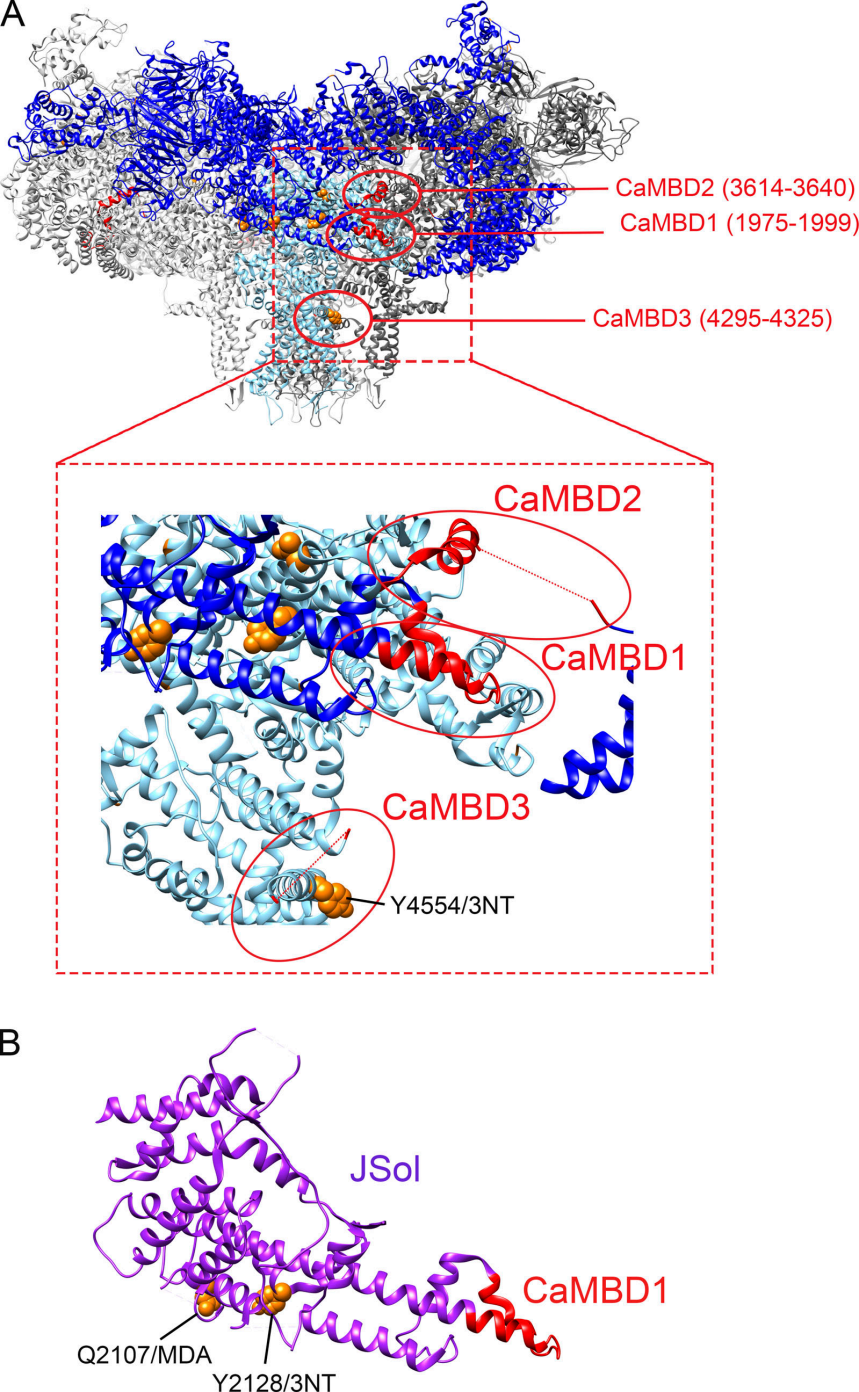
**Oxidative modifications proximal to the CaM binding sites on RyR1. (A)** Overview of three proposed CaMBDs in perspective to the RyR1 homotetramere structure as previously described (CaMB1, residues 1975–1999; CaMB2, residues 3614–3640; CaMB3, residues 4295–4325) (Yuchi and Van Petegem, 2016; Lau et al., 2014). **(B)** Oxidative PTM Y4554/3-NT was localized in proximity to the proposed CaMBD3. PTMs were identified with mass spectrometry, the models were generated with UCSF-Chimera using PDB-EBI protein model 7m6a (Melville et al., 2022).

**Figure S5.**
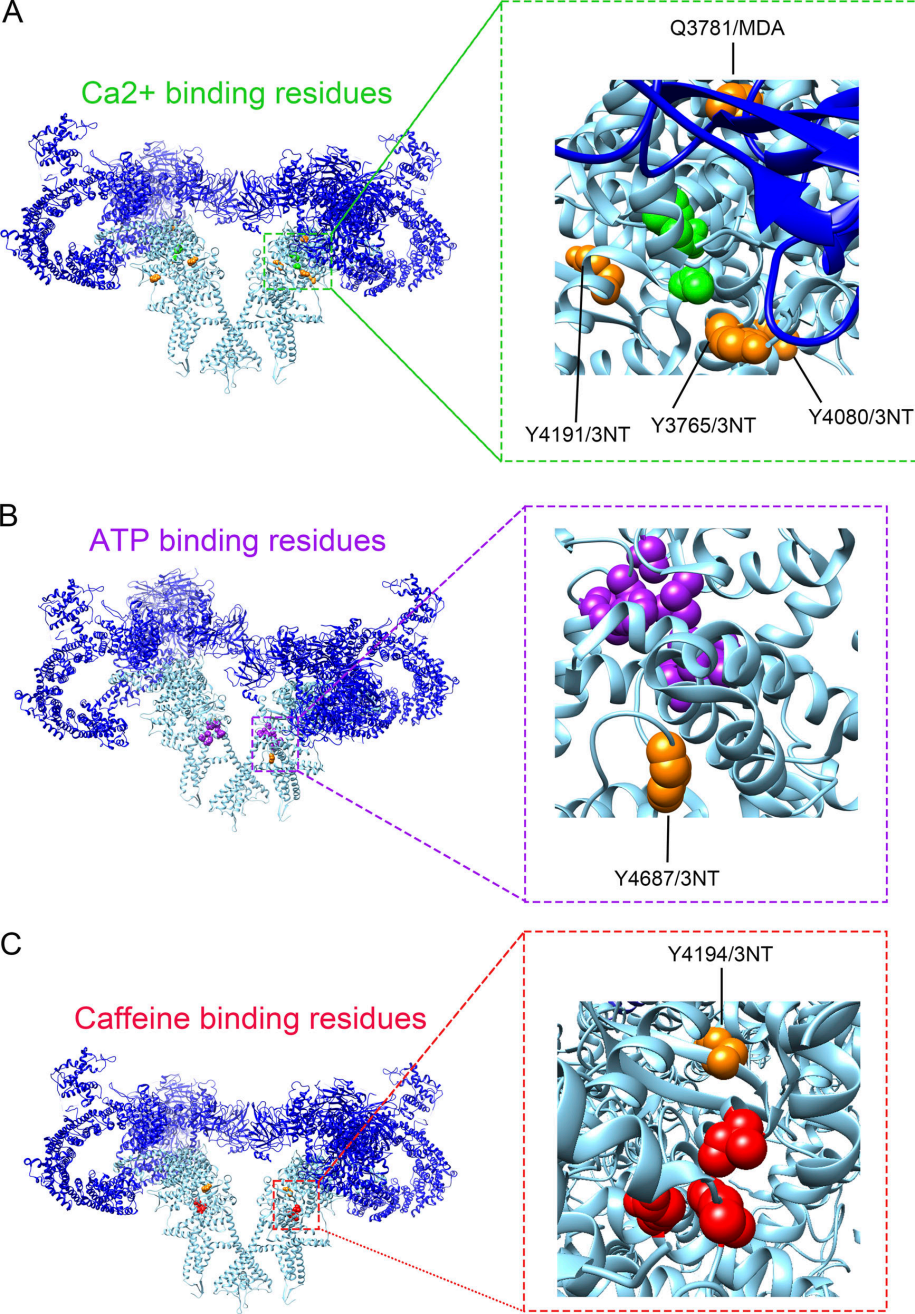
**3-NT and MDA modifications in relation to the binding residues for Ca^2+^, ATP and caffeine in the core of RyR1.** 3-NT and MDA modifications are depicted as spheres in orange. **(A)** Y4194/3NT, Y3765/3NT, Y4090/3NT and Q3781/MDA in relation to the Ca^2+^ binding residues shown in green: E3893, E3967, T5001. **(B)** Y4687/3NT in relation to the ATP binding residues shown in purple: M4954, F4959, T4979, L4985, K4211, K4214, K4215 and E4955. **(C)** Y4194/3NT in relation to the caffeine binding residues shown in red: W4716, I4996 and E4239. PTMs on RyR1 were identified with mass spectrometry after incubation of crude SR with SIN-1 ([10 mM], 15 min at RT, *n* = 3). The models were generated with UCSF-Chimera using PDB-EBI protein model 7m6a (Melville et al., 2022).

**Figure S6.**
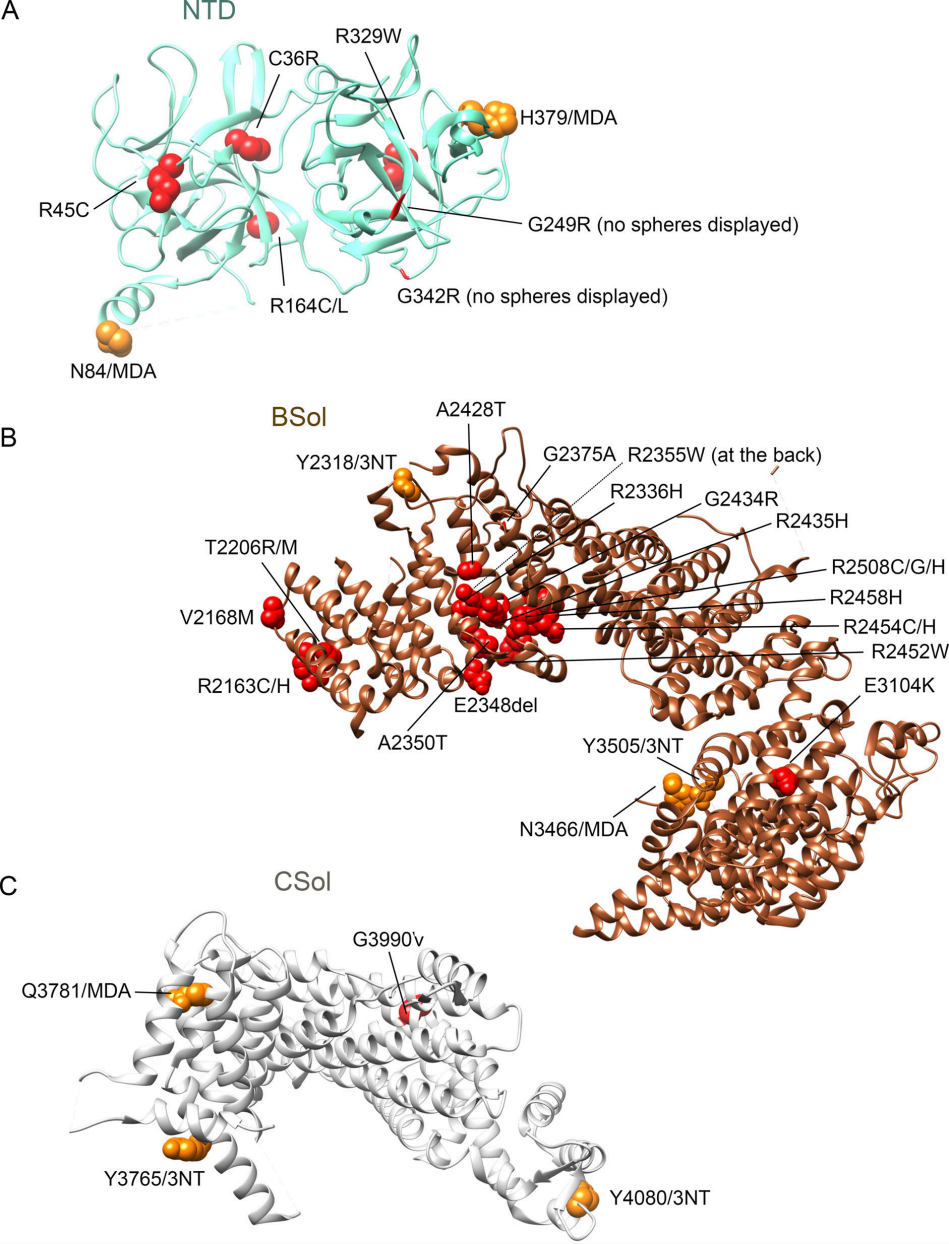
**Overlay of residues found to be mutated in malignant hyperthermia and the identified oxidative post-translational modifications.** The RyR1 three-dimensional model is adapted from the 7m6a cryo-EM structure and generated with UCSF-Chimera (Melville et al., 2022) and the location of the modifications was determined by Clustal protein sequence alignment of the mouse and rabbit protein sequence of RyR1. Red speres and ribbon marks resides know to be diagnostic for malignant hyperthermia (MH) and hence has a gain-of-function phenotype. Amino acids on the left of the number is the original RyR1 residue and MH diagnostic mutation on the right side. Residues with more than one known mutation are separated by “/.” Orange colored spheres marks residues with 3-NT and MDA post-translational modifications. Alanine (A), Arginine (R), Asparagine (N), Cysteine (C), Deletion (del), Glutamic acid (E), Glutamine (Q), Glycine (G), Histidine (H), Leucine (L), Methionine (M), Threonine (T), Tryptophan (W), Tyrosine (Y), Valine (V).

Fig. S1 shows simplified chemical reactions for the formation of 3-NT and MDA modifications. Figs. S2, S3, S4, S5, and S6 show alternative views of the RyR1 structure, oxPTMs, and regulatory units. Table S1 lists all oxidative PTMs found on RyR1 from mouse skeletal muscle crude SR incubated in SIN-1 (and their relative location in the rabbit 7ma6 model of RyR1). Table S2 shows clustalW sequence alignment between mouse (upper row) and rabbit (lower row) RyR1. The sequence homology between mouse and rabbit RyR1 is 95.94%.

## Results

### SIN-1 enhances RyR1 open probability

The effect of ONOO^•−^ on RyR1 channel activity was assessed by channel recordings in the absence and presence of the ONOO^•−^ donor SIN-1. SIN-1 is a metabolite of molsidome that spontaneously releases NO and O_2_^•−^ which react to form ONOO^•−^ under physiological conditions (Martin-Romero et al., 2004; Hogg et al., 1992). The recordings were performed 15 min after application of SIN-1 (1 mM) as that was the minimum time required to generate a significant increase of ONOO^•−^-induced PTMs on RyR1, assessed with 3-NT accumulation (Fig. 1 A). SIN-1 induced an approximately threefold increase in RyR1 channel P_o_ as compared with the control condition (vehicle) (Fig. 1, B and D). SIN-1 has been reported to modify RyR1 cysteines through thiol oxidation in addition to S-nitrosylation (Xu et al., 1998). Since cysteine thiol modification is known to enhance RyR1 channel activity (Sun et al., 2003; Xu et al., 1998), the channel preparation was preincubated with the reducing agent DTT (1 mM) to protect cysteine thiols from oxidation to –S-S- groups, from electron withdrawal (Cleland, 1964; Eager et al., 1997), and from *S*-nitrosylation (Jin et al., 2012). There was still a robust SIN-1 effect on RyR1 P_o_ in the presence of DTT (Fig. 1 C) but slightly blunted as compared with no DTT (Fig. 1 D). Furthermore, the SIN-1 effect on RyR1 channel activity appears dose-dependent, as P_o_ increased by ∼30% when SIN-1 concentration increased from 0.2 to 1 mM, after which the effect saturated. This increase was of the same magnitude both in the absence and presence of DTT (Fig. 1 E). RyR1 activity is dependent on *cis* Ca^2+^ concentration ([Ca^2+^]_cyt_) with activation enhanced by low [Ca^2+^]_cyt_ (∼nM–µM, by binding to specific high-affinity Ca^2+^ sites) (Meissner et al., 1986; Rodney et al., 2000). Here, SIN-1 induced significant changes in P_o_ at both 1 and 100 nM [Ca^2+^]_cyt_, which implies that the SIN-1 effect has no direct pronounced Ca^2+^ dependence (Fig. 1 F).

### Oxidative PTMs on RyR1 residues identified with MS

**Figure 2.**
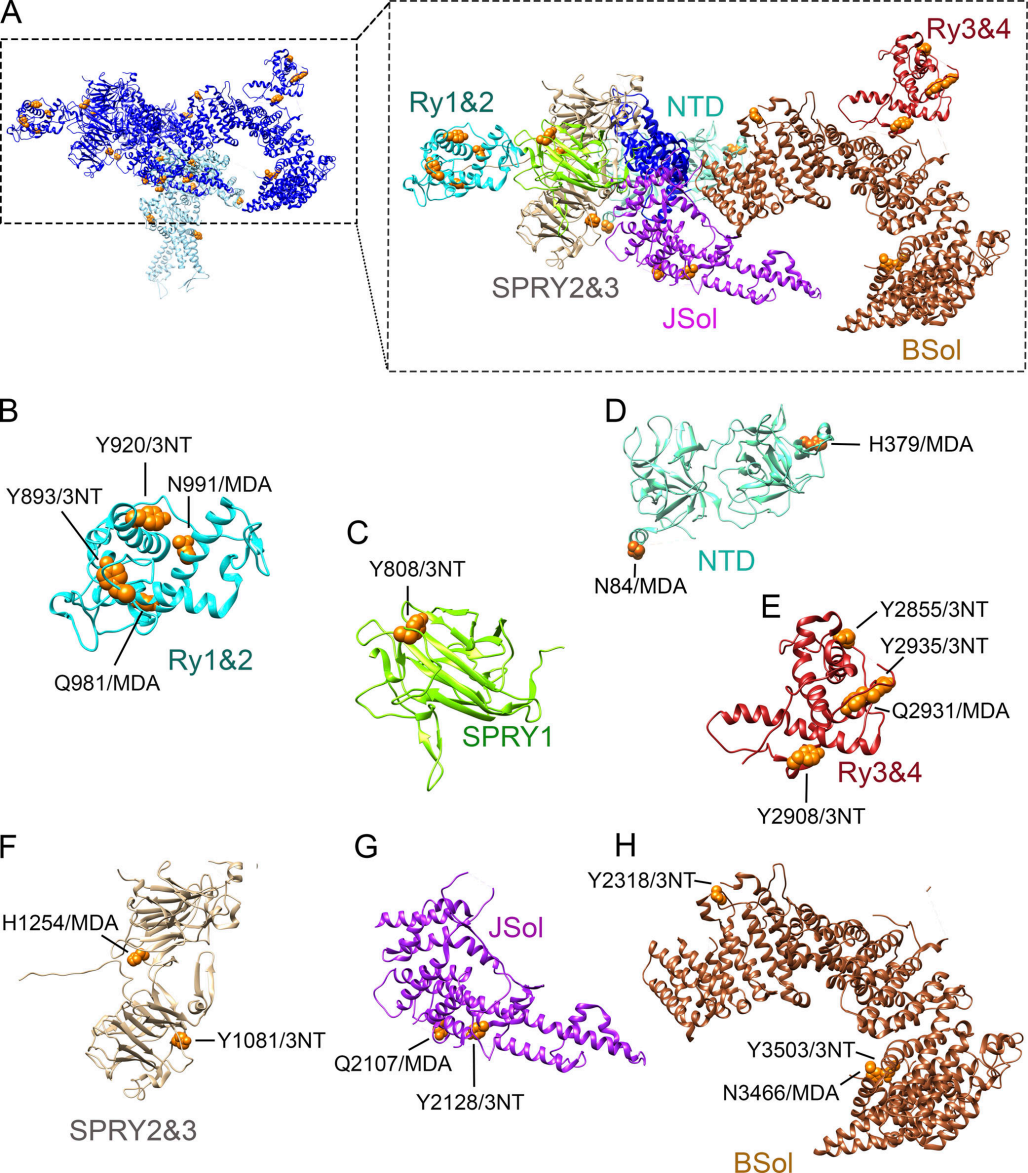
**Oxidative modifications localized to the domains in the cytosolic shell of RyR1. (A)** Overview of one RyR1 monomer with the oxidative PTM induced by SIN-1 in the cytosolic shell with coloring of the different domains, which were kept consistent throughout the figures. The RyR1 cryo-EM structure was adapted from Melville et al. (2022) (PDB-EBI 7m6a) and the location of the modifications was determined by Clustal protein sequence alignment of the mouse and rabbit protein sequence of RyR1. (**B–H)** Tyrosine residues (Y) were nitrated (3-NT) and histidine (H), glutamine (Q), and asparagine (N) were MDA modified. 3-NT and/or MDA modifications were observed in RY1&2 (B), SPRY1 (C), NTD (D), Ry3&4 (E), SPRY 2&3 (F), JSol (G), and BSol (H). The 3-NT and MDA oxidative modifications were identified by mass spectrometry on RyR1 from mouse skeletal muscle crude SR that had been incubated in SIN-1 ([10 mM], 15 min at RT, *n* = 3, *N* = 8). The models were generated with UCSF-Chimera (Pettersen et al., 2004).

MS analysis was carried out on crude SR to identify which RyR1 amino acids were modified by SIN-1 treatment and to get a clearer understanding of the link between stable oxidative PTMs and RyR1 P_o_. The MS analyses were performed in the presence of DTT (1 mM), hence disulfide bonds were reduced and possible RyR1 S-nitrosylation on RyR1 was reduced. Two types of stable SIN-1–induced oxidative PTMs were identified on RyR1 by MS: 3-NT and MDA. Of the 5035 amino acid residues per RyR1 monomeric subunit, a total of 30 were identified with either a 3-NT or MDA modification ([SIN-1, 10 mM], 15 min incubation at RT, *n* = 3) (Table S1). The 3-NT and MDA modifications were present in several RyR1 domains (Fig. S2 A) but clustered only to a few specific regions. Thus, selectivity for the targeted residues on RyR1 is not apparent. As oxidative 3-NT and MDA modifications are added by non-enzymatic mechanisms (Radi, 2013; Bayden et al., 2011), the clustering of the modifications was somewhat unexpected. However, it also aligns with our previous findings on actin (Steinz et al., 2019). Of the 30 amino acids with oxidative PTMs, 18 were localized to the cytosolic shell and 8 in the core region of RyR1 (Fig. 2, B and C). The position of the four additional residues was not possible to specify as the resolution of these regions is not high enough to visualize them in the 7ma6 three dimensional structure of RyR1 (Melville et al., 2022).

### Oxidative 3-NT and MDA modifications were clustered to regulatory domains within the cytosolic shell and core of RyR1

Fig. 2 A displays the cytosolic shell of one RyR1 monomer and the modifications localized to each cytosolic domain found on RyR1 pretreated with SIN-1. Fig. 2, B–H, highlights the modifications in each domain, where two modifications (N84/MDA and H379/MDA, Fig. 2 D) were identified in the N-terminal domain (NTD). NTD is important for domain–domain interaction and is known for being a disease hotspot. This region is subject to 20 genetic variations that underlie several RyR1-related diseases, including MH, central core disease, and multiminicore disease (Kimlicka et al., 2013). All 20 mutations are associated with a gain-of-function phenotype and thus facilitate channel opening (Kimlicka et al., 2013). Here, the MDA-modified H379 is in close proximity with residue H383, which is a known disease mutation site (Kimlicka et al., 2013).

A cluster of 3-NT and MDA modification were within the RyR repeat (Ry1–4) domains (Fig. 2, B and E) and splA kinase and RyR (SPRY1 and 2&3) domains (Fig. 2, C and F). There are four RyR repeats within RyR1, with repeat Ry1&2 appearing between SPRY1 and SPRY2&3, whereas Ry3&4 are located within the Bridge Solenoid (BSol). While far apart in the sequence (Fig. S2 A), Ry1&2 and Ry3&4 appear close in the 3-D structure (Fig. 2 A). Modifications Y893/3NT, Y920/3NT, Q981/MDA, and N991/MDA were identified in RY1&2 and Y2849/3NT, Y2855/3NT, Y2908/3NT, Q2931/MDA, and Y2935/3NT in Ry3&4 (Fig. 2, C and F). Ry3&4 domain is known for its inherent phosphorylation sites that have been shown to enhance channel activation (Yuchi and Van Petegem, 2016). This includes the PKA phosphorylation site S2843 in RyR1 (S2808 in the cardiac isoform RyR2), and here, we show that adjacent residues in Ry3&4, including Y2849 (Fig. S2 A and Table S1 structural 3-D-position not known) and Y2855 (Fig. 2 E), were oxidatively modified.

SPRY domains are known to mediate protein–protein interactions. Mutation experiments in the loop formed by residues 671–681 of RyR1 have confirmed that SPRY1 (residues 628–849) is involved in binding to FKBP12 (Yuchi and Van Petegem, 2016; Yuchi et al., 2015). However, the precise SPRY1 domain residues involved are yet to be determined. Moreover, it also is plausible that additional residues are involved in FKBP12 binding. In SPRY1, we identified a 3-NT modification on Y808 (Fig. 2 F). SPRY2&3 (found within residues 1055–1656) contains the so-called divergent region two (DR2, residues 1298–1431), which have been shown to be important for skeletal muscle E–C coupling. For example, deletion or substitution with the cardiac RyR2 isoform of these residues resulted in loss of E–C coupling (Yamazawa et al., 1997; Perez et al., 2003). Two oxidative PTMs were identified in the SPRY2&3 (Y1332/3NT, Q1401/MDA, Fig. 2 F) and both clustered within the DR2 residues.

Oxidative 3-NT and MDA modifications were also present in the activation core of RyR1 (Fig. S3, A–E). Specifically, N3651/MDA and Y3657/3NT in the shell-core linker peptide domain (SCLP) (Fig. S3 B), Y3765/3NT, Q3781/MDA, and Y4080/3NT in the core solenoid domain (CSol, Fig. S3 C), Y4195/3NT in the thumb and forefingers domain (TaF, Fig. S3 D), and Y4554/3NT and Y4687/3NT the pseudo voltage sensor domain (pVSD, Fig. S3 E). Of these domains, the pVSD is thought to play a role in RyR1 gating through allosteric conformational changes of the six transmembrane helices of the pore (Yuchi and Van Petegem, 2016). Two 3-NT modifications were found on residues close to these helices: Y4687/3NT and Y4554/3NT (Fig. S3 E).

The modified pVSD residue Y4554/3NT is also adjacent to one of the binding domains of CaM (Fig. S4 A). CaM is a known modulator of RyR1 channel activity in a [Ca^2+^]_cyt_-dependent manner (Lanner et al., 2010): CaM activates RyR1 at low [Ca^2+^]_cyt_ and inhibits the channel at high [Ca^2+^]_cyt_ (Rodney et al., 2000). The specific CaM binding sites have not been determined with high resolution, but three CaM binding domains (CaMBD) have been described for RyR1: CaMBD1 (residues 1975–1999 in the JSol); CaMBD2 (residues 3614–3640 in the BSol/SCLP), and CaMBD3 (within residues 4295–4325, adjacent to the transmembrane helices) (Lau et al., 2014; Yuchi and Van Petegem, 2016). Moreover, the cytosolic shell JSol domain (residues 1657–2144) encompasses CaMBD1, and two modifications were identified in its proximity—Q2107/MDA and Y2128/3NT (Fig. S4 B).

### Peroxynitrite induced FKBP12 dissociation from RyR1

**Figure 3.**
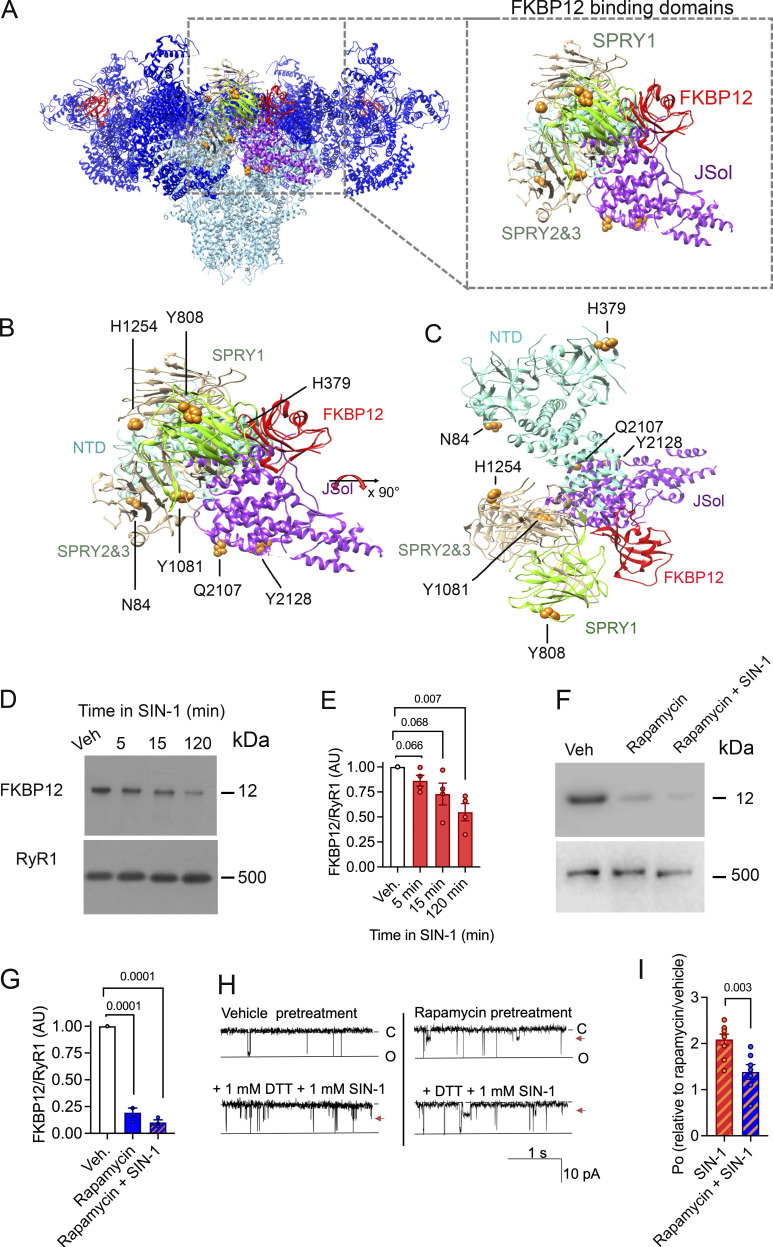
**MDA and 3-NT modifications of RyR1 results in FKBP12 dissociation. (A)** Visualization of the RyR1 homeotetramer with SIN1-induced oxidative 3-NT and MDA modifications found in domains that are proposed to be involved in the binding of FKBP12 (i.e., the NTD, SPRY1, SPRY3, and JSol domain) (Yan et al., 2015). **(B and C)** Magnification and (C) 90° x-axis turned magnification of the oxidative PTMs on RyR1 in perspective with the binding domains for FKBP12. N84/MDA, H379/MDA in NTD, Y808/3NT in SPRY1, Y1081/3NT, H1254/MDA in SPRY2/3, and Q2107/MDA and Y2128/3NT in the JSol domain. PTMs were identified with mass spectrometry, the models were generated with UCSF-Chimera (Pettersen et al., 2004) using PDB-EBI protein model 3J8H (Yan et al., 2015). **(D and E)** Immunoblot (D) and corresponding bar graph (E) of FKBP12 normalized to RyR1 of crude SR incubated in SIN-1 (1 mM). (Data = mean ± SEM, *n* = 4 for each condition, across four immunoblots). Approximate molecular weights are to the left of immunoblots. P = 0.066 between vehicle and 5 min, P = 0.068 between vehicle and 15 min, and P = 0.007 between vehicle and 120 min in 1 mM SIN-1. **(F and G)** Immunoblot (F) and corresponding bar graph (G) of FKBP12 normalized to RyR1 of crude SR incubated in rapamycin (5 mM) or rapamycin and SIN-1 [1 mM]. (Data = mean ± SEM, *n* = 3 for each condition, across four immunoblots). Approximate molecular weights are to the left of immunoblots. P = 0.0001 between vehicle and rapamycin and between vehicle and rapamycin +SIN-1. **(H)**. Representative RyR1 channel recordings showing RyR1 responses to DTT and 1 mM SIN-1 after pretreatment with rapamycin to dissociate FKBP12 (or vehicle). Channel opening is downward from zero current (C, solid line) to maximum open conductance (O, dashed line) at −40 mV. P_o_ of individual channel traces are 0.04 (vehicle pretreatment), 0.09 (vehicle pretreatment + DTT + SIN1), 0.07 (rapamycin pretreatment), and 0.082 (rapamycin pretreatment + DTT + SIN-1. Arrowhead indicates presence of substate activity. **(I)** Relative P_o_ (mean ± SEM, *n* = 8) from measurements of P_o_ at +40 and −40 mV showing RyR1 responses to DTT and 1 mM SIN-1 after pretreatment with rapamycin pretreatment to dissociate FKBP12 (or vehicle). Channel activity is presented as relative to that recorded after rapamycin or vehicle treatment, P = 0.003. Source data are available for this figure: SourceData F3.

High-resolution structural analyses (Yan et al., 2015; Yuchi et al., 2015) suggest that SPRY1 together with NTD, SPRY2&3, and JSol form the cleft in which FKBP12 binds (Fig. 3 A). Specifically, RyR1 residues 1687, 1780, and 1781 were proposed to be involved in the interaction (Yan et al., 2015). Our MS analyses of RyR1 exposed to SIN-1 (10 mM, 15 min) identified oxidative modifications N84/MDA (NTD), H379/MDA (NTD), Y808/3NT (SPRY1), Y1081/3NT (SPRY2&3), H1254/MDA (SPRY2&3), Q2107/MDA (Jsol), and Y2128/3NT (JSol), which were localized to this cleft region (Fig. 3, B and C).

Although the identified 3-NT and MDA modifications did not overlap directly with the proposed FKBP binding residues (Yan et al., 2015), immunoblotting experiments show that they result in FKBP12 dissociation from RyR1 (Fig. 3, D–E). RyR1 exposed to SIN-1 for 15 min resulted in ∼25% reduction of FKBP bound to RyR1, and ∼50% dissociation after 120 min. This aligns with the increase in RyR1 P_o_ after SIN-1 exposure (Fig. 1) as loss of FKBP12 binding to RyR1 is known to increase the P_o_ of the channel (Bellinger et al., 2008).

In a series of single-channel experiments, channels were pretreated with 5 μM rapamycin to dissociate FKBP12 from RyR1 (Ahern et al., 1997b; Richardson et al., 2017) prior to treating with 1 mM SIN-1 in the presence of DTT (Fig. 3, F–I). While SIN-1 activated FKBP12-dissociated channels, the response was significantly blunted at 1 mM SIN-1 when compared to channels that were pretreated with vehicle. That rapamycin pretreatment did not completely block SIN-1 from any activation effect may be explained by the incomplete removal of FKBP by rapamycin. However, overall, this result suggests that FKBP12 may play a role, at least in part, as a mediator of the increased channel activity included by SIN-1.

## Discussion

RyR1 has a key role in mediating SR Ca^2+^ release and enabling E–C coupling. Thus, RyR1 is a topic of great interest with regard to understanding E–C coupling and its potential as a target to counteract muscle dysfunction and weakness (Lanner et al., 2010; Waning et al., 2015; Bellinger et al., 2009). Several studies have shown that *S*-nitrosylation, DNP, and phosphorylation PTMs on RyR1 alter the channel activity. Moreover, excessive RyR1 PTMs are associated with FKBP12 dissociation, increased channel P_o_, and SR Ca^2+^ leak. This can result in reduced SR Ca^2+^ load and Ca^2+^ release during E–C coupling and, ultimately, impaired muscle contractility (Andronache et al., 2009; Santulli et al., 2017; Lanner et al., 2010; Waning et al., 2015; Bellinger et al., 2009). Immunoblotting has, thus far, been the most frequently used method to assess RyR1 PTMs, which reflects the level of modifications that the antibody of interest detects. However, there is limited information on which RyR1 residues are subject to these modifications. In fact, there are only a few specific RyR1 residues with PTMs that have directly been linked to altered RyR1 gating, regulation, or channel function (Moore et al., 1999; Chaube et al., 2014; Sun et al., 2003). For example, *S*-nitrosylation of Cys3635 can activate RyR1 (Sun et al., 2003) and phosphorylation of Ser2843 was shown to enhance RyR1 activity by causing dissociation of FKBP12 from RyR1 (Stange et al., 2003; Jacko et al., 2018; Reiken et al., 2003; Rullman et al., 2013; Lanner et al., 2010). Nevertheless, it has been unclear how stable oxidative 3-NT and MDA modifications affect RyR1 channel activity.

While the NO and O_2_^•−^-generating compound SIN-1 has been shown to induce RyR2 oxidation, interdomain destabilization, FKBP12.6 dissociation, and RyR2 Ca^2+^ (Yano et al., 2005), this is the first time that SIN-1 has been shown to induce oxidative 3-NT and MDA modifications on RyR1. These modifications result in an increased channel P_o_. With MS, we identified which RyR1 residues exhibited oxidative 3-NT and MDA modifications. Modifications were mapped into the cryo-EM reconstructed RyR1 structure (PDB ID 7m6a) (Melville et al., 2022) and found to be present in the cytosolic shell as well as the core.

As our results are from mice and the 7m6a model originates from rabbit RyR1, Clustal was used to align mouse and rabbit RyR1 amino acid sequences, which confirmed a 96% homology between the two species (Table S2). Nearly all 3NT/MDA modified amino acid residues that were mapped in the 7m6a protein structure were in homologous regions. The modified residues which were close to amino acids that were not homologous are listed in Table S2. Importantly, none of the non-homologous amino acids were charged residues (arginine [R], lysine [K], aspartate [D], glutamate [E], or histidine [H]), which could have attracted adjacent residues to be more prone to 3-NT and/or MDA mutations. Thus, the similarity in RyR1 amino acid sequence between mouse and rabbit implies that the rabbit 7ma6 structure is a representative scaffold for mapping RyR1 modifications obtained from mouse muscle.

Several of the modified residues were in domains known for their regulatory function of the channel. For instance, 3-NT and MDA modifications were present in SPRY1, NTD, SPRY2&3, and JSol (Fig. 3), which form the cleft where the acknowledged channel modulator FKBP12 binds. Here, we showed that 3-NT and MDA modifications on RyR1 induced by SIN-1 resulted in the dissociation of FKBP12 from RyR1. Additional RyR1 channel regulators affecting channel behavior include CaM, Ca^2+^, caffeine, and ATP, with simultaneous binding of these regulators increasing RyR1 P_o_ (des Georges et al., 2016; Chirasani et al., 2021). We identified oxidative PTMs adjacent to the CaMBD (Fig. S4), and a limited number of 3-NT or MDA modifications were identified in direct proximity to residues that have been described to bind Ca^2+^, caffeine, or ATP to RyR1 (Fig. S5) (des Georges et al., 2016; Melville et al., 2022). Fig. S5 A shows that Y3765/3NT, Y4080/3NT, and Y4194/3NT are neighboring the Ca^2+^ binding residues and that Y4687/3NT and Y4194/3NT were close to the ATP binding and caffeine binding residues, respectively (Fig. S5, B and C). Here, the effect of bulky 3-NT and MDA PTMs on small effectors affinity has not been explored, which is a limitation and needs further investigation. Future studies would also benefit from exploring the dose dependency of 3-NT and MDA PTMs on RyR1 and its effect on gating, as increases in P_o_ were observed in the presence of 1 mM SIN-1, whereas MS analyses were performed with 10 times higher concentration. Furthermore, added PTMs can lead to allosteric changes and alteration in the local chemical environment which can, in turn, change binding affinity (e.g., FKBP12). A single 3-NT or MDA modification on a residue (or close by proximity) that is known to cause gain-of-function mutations may potentially also directly lead to alterations in RyR1 P_o_. In comparison, a single gene variation in the *RYR1* gene can directly alter the channel function and cause a gain-of-function phenotype, which is the case in the RYR1-related disease MH (Mader et al., 2020). There are ∼50 gene variants of *RyR1* that have been acknowledged as diagnostic mutations by The European Malignant Hyperthermia Group (Mader et al., 2020). Here, we overlayed the amino residues of known MH causative *RyR1* gene variations (Mader et al., 2020) and our oxidative 3-NT and MDA modifications in the 7ma6 structure. Although no known MH-causative residues directly overlapped with the observed 3-NT and MDA modifications, there were several located within the same domain (Fig. S6), for instance, in BSol that contains several residues linked to MH (Fig. S6 B), NTD (Fig. S6 A), and CSol (Fig. S6 C).

**Figure S7.**
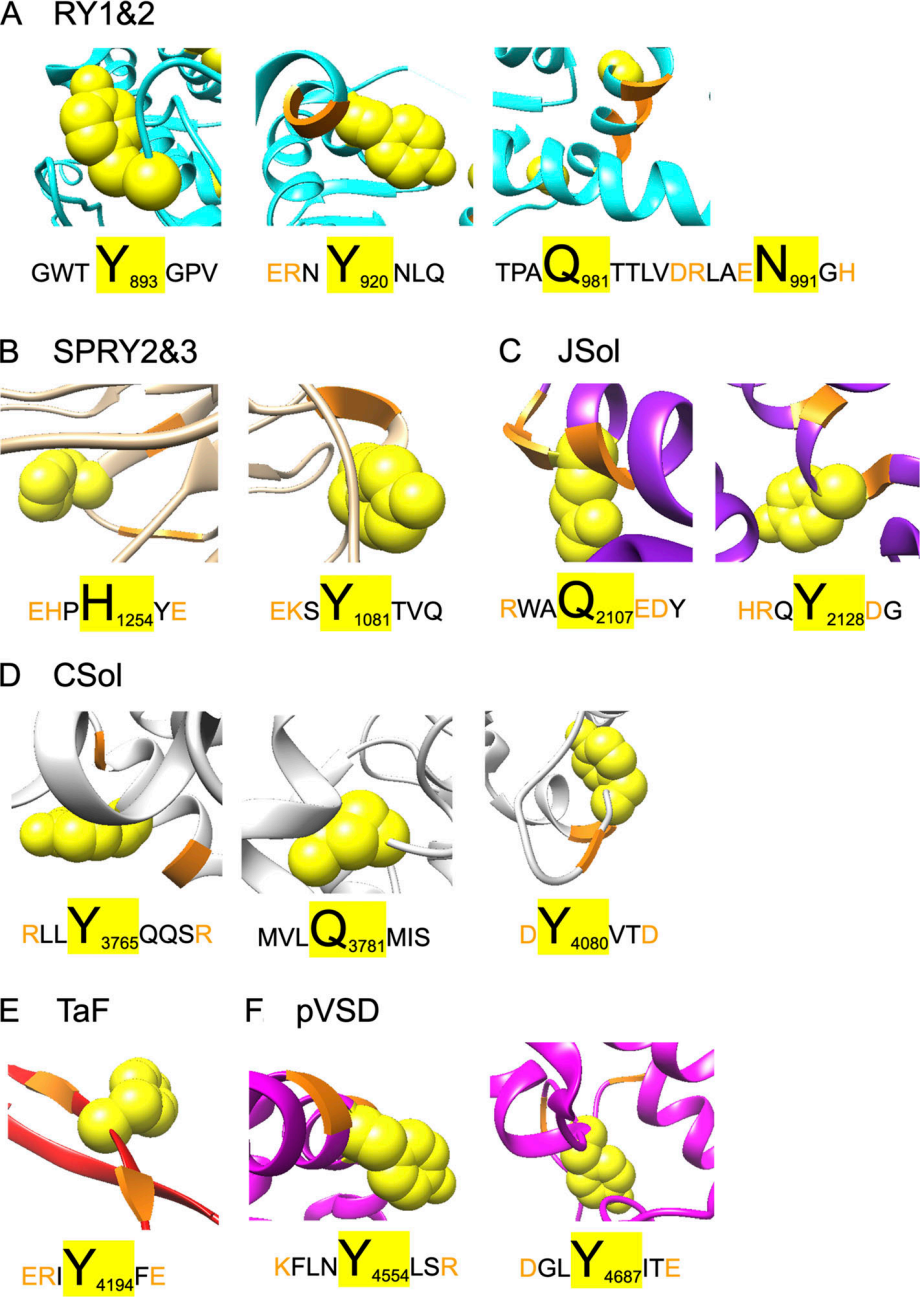
**3-NT and MDA modifications of RyR1 that are surrounded by amino acids with an electrically charged side chain and/ or that located in a loop structure.** The RyR1 three-dimensional medel is adapted from the 7m6a cryo-EM structure of PDB-EBI (Melville et al., 2022) and the location of the modifications was determined by Clustal protein sequence alignment of the mouse and rabbit protein sequence of RyR1. **(A–F)** The modifications that are shown in the domains RY1&2 (A), SPRY2&3 (B), JSol (C), CSol (D), TaF (E), pVSD (F) are either tyrosine residues (Y) that were nitrated (3-NT) or histidine (H), glutamine (Q) or asparagine (N) residues that were MDA modified and are all shown as yellow spheres. Charged amino acid residues, e.g. Arginine (R), Lysine (K), Aspartic Acid (D), Glutamic Acid (E) and Histidine (H), are marked in yellow. The shown sequence and amino acid identifier (e.g., 85 for N85) are conform the RyR1 sequence of *Mus musculus*.

3-NT and MDA are introduced by non-enzymatic reactions, hence, the selectivity for residues that will be affected is not apparent and there is currently no consensus regarding the selectivity of oxidative modifications. However, the local microenvironment and the structural features of the protein are thought to affect the selectivity. Moreover, residues located near charged amino acids or on loop structures appear more prone to modification (Radi, 2013; Bayden et al., 2011; Souza et al., 1999; Steinz et al., 2019). This is consistent with our results, where a majority (23 out of 30) of the modified residues (tyrosine, histidine glutamine and asparagine) were flanked by charged amino acids (i.e., arginine [R], lysine [K], aspartic acid [D], glutamic acid [E], or histidine [H]) and/or in loop structures as exemplified in Table S1 and Fig. S7. With molecular dynamics simulations, we have previously shown that MDA (C_3_H_3_O) and 3-NT (NO_2_) on actin obstructs hydrogen bonds and chemical contacts in the local environment (Steinz et al., 2019), which creates local steric restrictions and triggers conformational changes. The 3-NT and MDA modifications on RyR1 supposedly have a similar effect: i.e., that the addition of bulky 3-NT and MDA modifications leads to allosteric alterations contributing to FKBP12 dissociation from RyR1 as observed by the immunoblot results (Fig. 3).

Here, we show that induced oxidative 3-NT and MDA modifications were directly linked to altered RyR1-mediated Ca^2+^ release. We have previously shown that muscle from mice with arthritis have higher levels of nitration markers on the RyR1–DHPR super complex which was accompanied by altered Ca^2+^ release (Yamada et al., 2015). In addition to RyR1, other proteins important for muscle contraction have been displayed with elevated 3-NT and MDA modifications in the skeletal muscle of diseased and aged subjects (Steinz et al., 2019; Kanski et al., 2005). This includes (1) skeletal muscle actin in patients and mice with rheumatoid arthritis which have direct negative effects on contractile force production and deleterious effects on actin polymerization (Steinz et al., 2019), (2) creatine kinase, tropomyosin, and myosin light chain in old rats (Kanski et al., 2005), and (3) the Ca^2+^ pump (SR Ca^2+^ ATPase, SERCA) in rat hearts after ischemia-reperfusion injury (Tang et al., 2010). Thus, these oxidative modifications should be considered important players in the continued efforts to understand RyR1 gating and its effects on cellular processes in both physiological and pathophysiological conditions. This will aid in the continued quest to develop pharmaceutical therapies to improve muscle function for patients afflicted with muscle weakness and fatigue.

## Supplementary Material

Table S1 All oxidative PTMs found on RyR1 from mouse skeletal muscle crude SR incubated in SIN-1 (*n* = 3 with pooled muscle from 12 mice in total) and their relative location in the rabbit 7ma6 model of RyR1.

Table S2 ClustalW sequence alignment between mouse (upper row) and rabbit (lower row) RyR1. The sequence homology between mouse and rabbit RyR1 is 95.94%.

SourceData F1 is the source file for Fig. 1.

SourceData F3 is the source file for Fig. 3.

## Data Availability

The data underlying Figs. 2 and 3 and supplemental material are openly available in Zenodo at https://doi.org/10.5281/zenodo.13373260.
