# Supplementary material for: Stable oxidative posttranslational modifications alter the gating properties of RyR1
Source: J Gen Physiol. 2024 Nov 5;156(12):e202313515. doi: 10.1085/jgp.202313515 (PMC11540854; doi:10.1085/jgp.202313515)
Supplement: Table S1 — All oxidative PTMs found on RyR1 from mouse skeletal muscle crude SR incubated in SIN-1 (n = 3 with pooled muscle from 12 mice in total) and their relative location in the rabbit 7ma6 model of RyR1. [file JGP_202313515_TableS1.docx]

| **No.** | **Domain abbreviation (residues)** | **Domain full name** | **3-NT (RyR1 *mus musculus*)** | **MDA *(*RyR1 *mus musculus)*** | **Residue in 7ma6 *(*RyR1 *oryctolagys caniculus)*** | **Adjacent Residues (RyR1 *mus musculus*)** |
| --- | --- | --- | --- | --- | --- | --- |
|  | **Cytosolic Shell  (1-3613)** |  |  |  |  |  |
| **1.** | **NTD-A  (1-208)** | N-terminal A |  | N85-MDA | N84 | **EMLANTVE** |
| **2.** | **NTD-B  (209-392)** | N-terminal B |  | H380-MDA | H379 | **AMLHQEG** |
| **3.** | **SPRY1  (628-849)** | SP1a/ Ryanodine Receptor domain 1 | Y809-3NT |  | Y808 | **PPGYAPC** |
| **4.** | **RY1&2  (850-1054)** | RyR Repeat 1/2 | Y894-3NT |  | Y893 | **GWTYGPV** |
| **5.** |  | RyR Repeat 1/2 | Y921-3NT |  | Y920 | **ERNYNLQ** |
| **6.** |  | RyR Repeat 1/2 |  | Q982-MDA | Q981 | **TPAQTTL** |
| **7.** |  | RyR Repeat 1/2 |  | N992-MDA | N991 | **LAENGHN** |
| **8.** | **SPRY2&3  (1055-1656)** | SP1a/ Ryanodine Receptor domain 2 | Y1082-3NT |  | Y1081 | **EKSYAVQ** |
| **9.** |  | SP1a/ Ryanodine Receptor domain 3 |  | H1255-MDA | H1254 | **EHPHYEV** |
| **10.** |  | SP1a/ Ryanodine Receptor domain 3 | Y1333-3NT |  | Y1332 (position unknown) | **DTDYENL** |
| **11.** |  | SP1a/ Ryanodine Receptor domain 3 |  | Q1402-MDA | Q1401  (position unknown) | **MMTQPPS** |
| **12.** | **JSol  (1657- 2144)** | Junctional Solenoid |  | Q2108-MDA | Q2107 | **RWAQEDF** |
| **13.** |  | Junctional Solenoid | Y2129-3NT |  | Y2128 | **HRQYDGL** |
| **14.** | **BSol  (2145-3613)** | Bridge Solenoid | Y2319-3NT |  | Y2318 | **AKGYPDI** |
| **15.** | **RY3&4  (2735-2938)** | Bridge Solenoid (RyR Repeat 3/4) | Y2850-3NT |  | Y2849  (position unknown) | **AQTYDPR** |
| **16.** |  | Bridge Solenoid (RyR Repeat 3/4) | Y2856-3NT |  | Y2855 | **REGYNPQ** |
| **17.** |  | Bridge Solenoid (RyR Repeat 3/4) | Y2909-3NT |  | Y2908 | **LVPYDTL** |
| **18.** |  | Bridge Solenoid (RyR Repeat 3/4) |  | Q2932-MDA | Q2931 | **KFLQMNG** |
| **19.** |  | Bridge Solenoid (RyR Repeat 3/4) | Y2936-3NT |  | Y2935 | **MNGYAVT** |
| **20.** |  | Bridge Solenoid |  | N3467-MDA | N3466 (position unknown) | **NEINNMS** |
| **21.** |  | Bridge Solenoid |  | Q3486-MDA | Q3485  (position unknown) | **GDVQSGG** |
| **22.** |  | Bridge Solenoid | Y3504-3NT |  | Y3503  (position unknown) | **GDRYSVQ** |
|  | **Channel and Activation Core (3614-5037)** |  |  |  |  |  |
| **23.** | **SCLP (3614-3666)** | Shell-Core linker Peptide |  | N3652-MDA | N3651 | **RACNMFL** |
| **24.** |  | Shell-Core linker Peptide | Y3658-3NT |  | Y3657 | **LESYKAS** |
| **25.** | **CSol (3667-4174)** | Core Solenoid | Y3768-3NT |  | Y3765 | **RLLYQQS** |
| **26.** |  | Core Solenoid |  | Q3784-MDA | Q3781 | **MVLQMIS** |
| **27.** |  | Core Solenoid | Y4083-3NT |  | Y4080 | **FQDYVTD** |
| **28.** | **TaF  (4175-4253)** | Thumb and Forefingers | Y4197-3NT |  | Y4194 | **ERIYFEI** |
| **29.** | **pVSD (4541-4819)** | Pseudo voltage sensor domain | Y4552-3NT |  | Y4554 | **FLNYLSR** |
| **30.** |  | Pseudo voltage sensor domain | Y4685-3NT |  | Y4687 | **DGLYITE** |

**Supplemental. Table. 1.** List of all oxidative PTMs found on RyR1 from mouse skeletal muscle crude SR incubated in SIN-1 (n=3 with pooled muscle from 12 mice in total) and their relative location in the rabbit 7ma6 model of RyR1. Oxidative modifications of RyR1 identified by mass spectrometry. The far-right column indicates the amino acids that were identified with either a 3-NT or MDA modification of RyR1 (orange) and the closely located to electrically charged amino acids residues (yellow), i.e. Arginine (R), Lysine (K), Aspartic acid (D), Glutamic acid (E) and Histidine (H), marked in yellow, one letter code and identifier.
