# Supplementary material for: Stable oxidative posttranslational modifications alter the gating properties of RyR1
Source: J Gen Physiol. 2024 Nov 5;156(12):e202313515. doi: 10.1085/jgp.202313515 (PMC11540854; doi:10.1085/jgp.202313515)
Supplement: Table S2 — ClustalW sequence alignment between mouse (upper row) and rabbit (lower row) RyR1. The sequence homology between mouse and rabbit RyR1 is 95.94%. [file JGP_202313515_TableS2.docx]

**H380-MDA (NTD-B)**Close to this residue in mouse we see an M instead of an I.
sp|E9PZQ0|RYR1_MOUSE AAPDPKALRLGVLKKKAMLHQEGHMDDALSLTRCQQEESQAARMIYSTAGLYNQFIKGLD 420
sp|P11716|RYR1_RABIT AAPDPKALRLGVLKKKAILHQEGHMDDALFLTRCQQEESQAARMIHSTAGLYNQFIKGLD 419
*****************:*********** ***************:**************

**Y1082-3NT (SPRY2&3)**Directly next to this residue in mouse we see an A instead of a T.
sp|E9PZQ0|RYR1_MOUSE SYAVQSGRWYFEFEAVTTGEMRVGWARPELRPDVELGADDLAYVFNGHRGQRWHLGSEPF 1140
sp|P11716|RYR1_RABIT SYTVQSGRWYFEFEAVTTGEMRVGWARPELRPDVELGADELAYVFNGHRGQRWHLGSEPF 1139
**:************************************:********************

**Y1332-3NT (SP1a/ Ryanodine receptor domain 3)**

Close to this residue in mouse we see a T instead of a P.
sp|E9PZQ0|RYR1_MOUSE EDEARAAEPDTDYENLRRSAGGWGEAEGGKDGTAKEGTPGGTAQAGVEAQPARAENEKDA 1380
sp|P11716|RYR1_RABIT EDEARAAEPDPDYENLRRSAGGWGEAEGGKEGTAKEGTPGGTPQPGVEAQPVRAENEKDA 1379
********** *******************:*********** * ******.********

**Q1402-MDA (SP1a/ Ryanodine Receptor domain 3)**

Close to this residue in mouse we see a S instead of an A.
sp|E9PZQ0|RYR1_MOUSE TTEKNKKRGFLFKAKKVAMMTQPPSTPALPRLPRDVVPADNRDDPEIILNTTTYYYSVRV 1440

sp|P11716|RYR1_RABIT TTEKNKKRGFLFKAKKAAMMTQPPATPALPRLPHDVVPADNRDDPEIILNTTTYYYSVRV 1439

****************.*******:********:**************************

**Q2108-MDA (Junctional Solenoid)**Close to this residue in mouse we see an F instead of a Y
sp|E9PZQ0|RYR1_MOUSE SHTVVRWAQEDFVQSPELVRAMFSLLHRQYDGLGELLRALPRAYTISVSSVEDTMSLLEC 2159

sp|P11716|RYR1_RABIT SHMVVRWAQEDYVQSPELVRAMFSLLHRQYDGLGELLRALPRAYTISPSSVEDTMSLLEC 2158

** ********:*********************************** ************

**Q3485-MDA (BSol)**

Directly next to this residue in mouse we see a V instead of an A.
sp|E9PZQ0|RYR1_MOUSE AKAGDVQSGGSDQERTKKKRRGDRYSVQTSLIVATLKKMLPIGLNMCAPTDQDLIVLAKA 3539
sp|P11716|RYR1_RABIT AKAGDAQSGGSDQERTKKKRRGDRYSVQTSLIVATLKKMLPIGLNMCAPTDQDLIMLAKT 3538
*****.*************************************************:***:

**Supplemental Table 2. Clustalw sequence alignment between mouse (upper row) and rabbit (lower row) RyR1.** The sequence homology between mouse and rabbit RyR1 is 95.94%, The listed alignments are 3-NT/ MDA modified residues (marked in yellow) that are in proximity of residues (shown in red) which differ between mouse and rabbit RyR1.
