# Supplementary figures and images for: Stable oxidative posttranslational modifications alter the gating properties of RyR1
Source: J Gen Physiol. 2024 Nov 5;156(12):e202313515. doi: 10.1085/jgp.202313515 (PMC11540854; doi:10.1085/jgp.202313515)

Source Blot RyR1 Fig 1

Time in SIN-1 (min)

Veh

1

5

15

120

3-NT

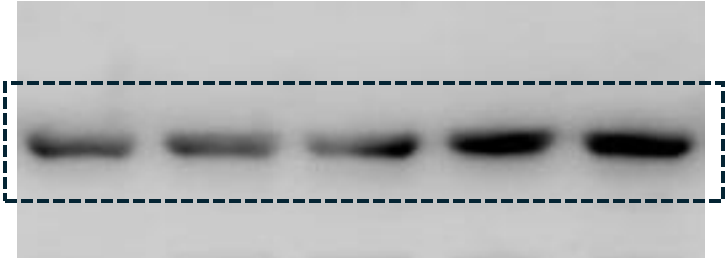

250 —

RyR1

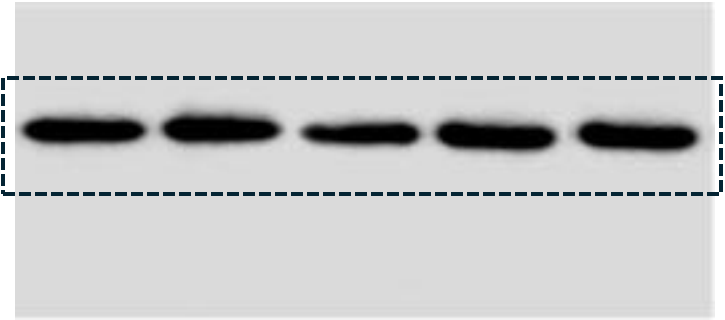

250 —

Supplement: SourceData F1 — is the source file for Fig. 1. [file JGP_202313515_SourceDataF1.pdf]

Source Blot Fig 3D

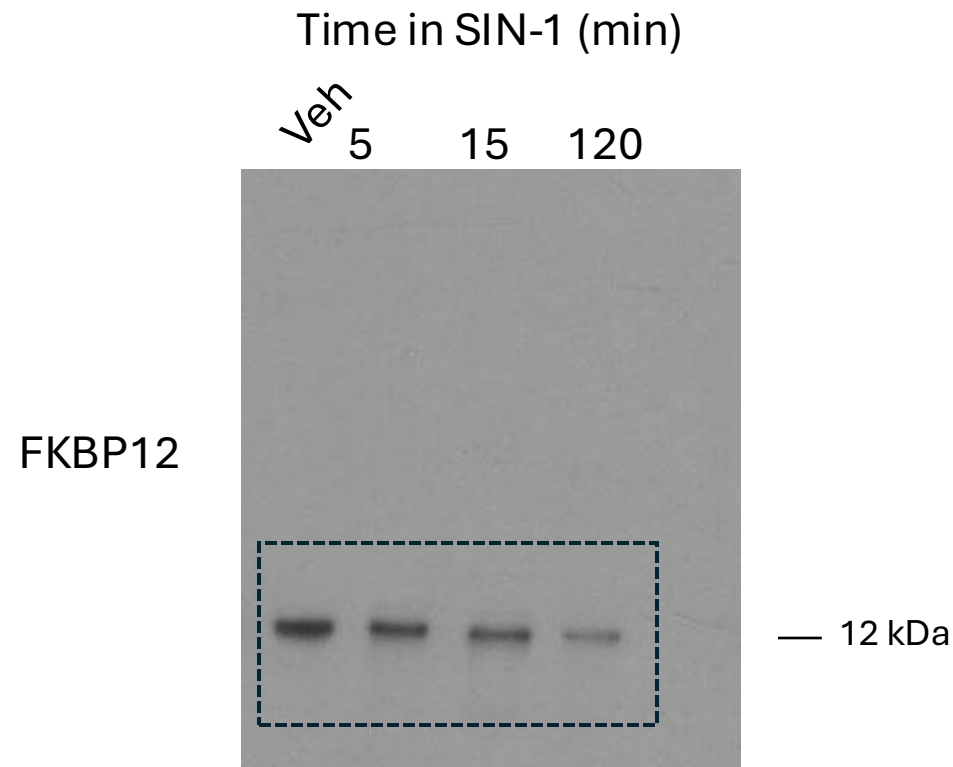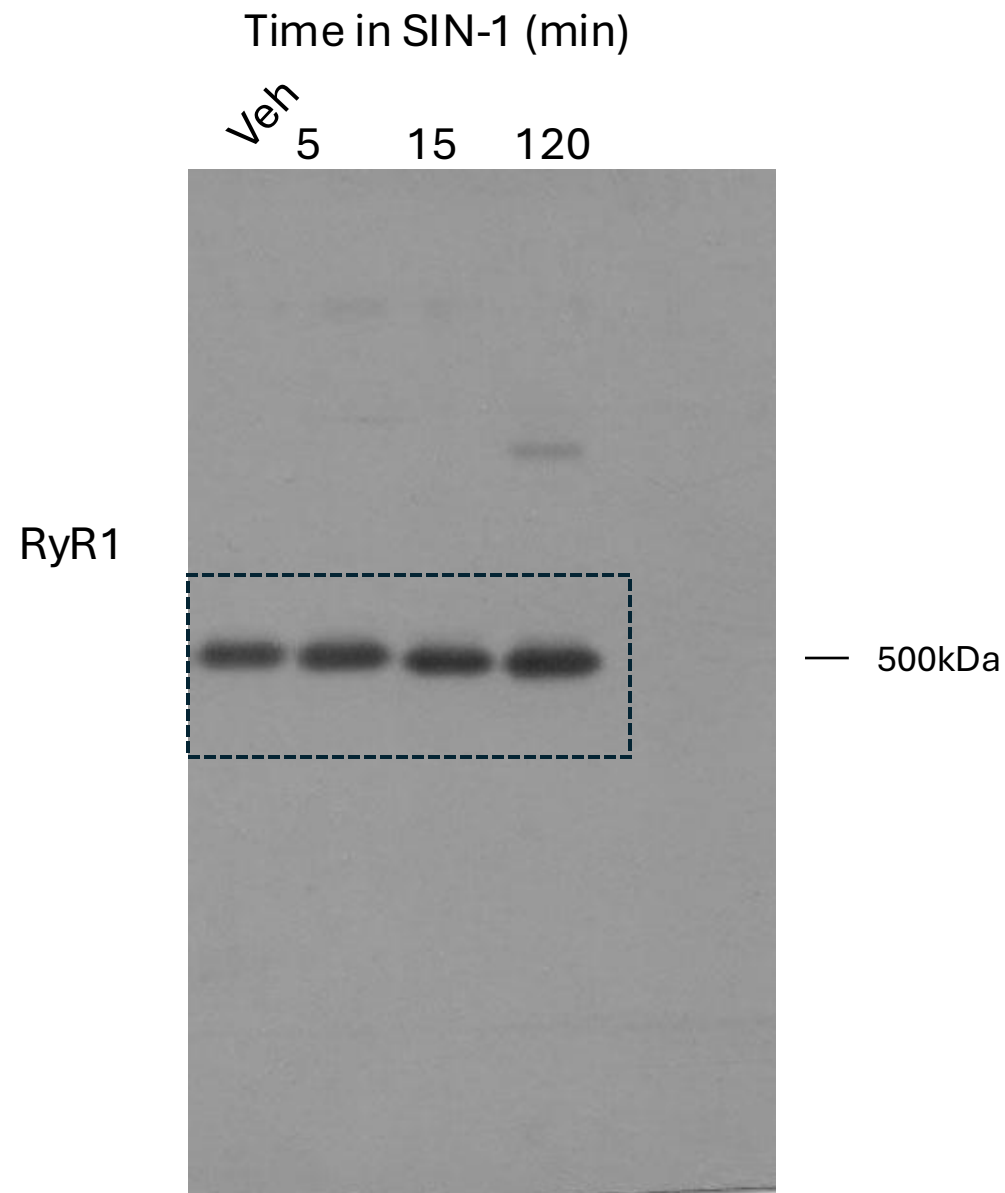

Source blot Fig. 3F

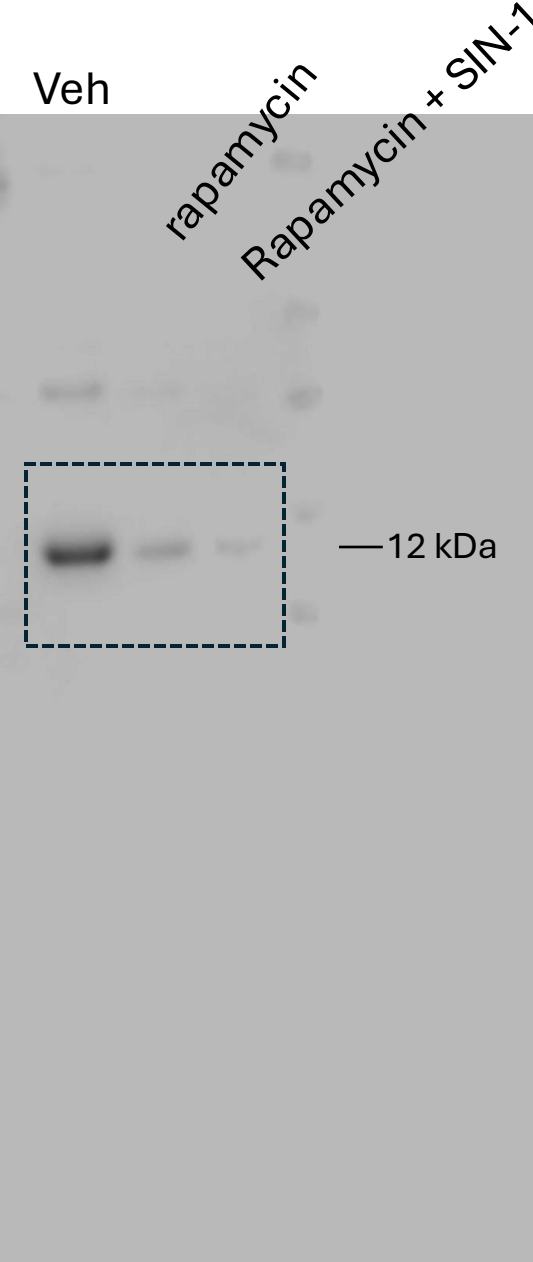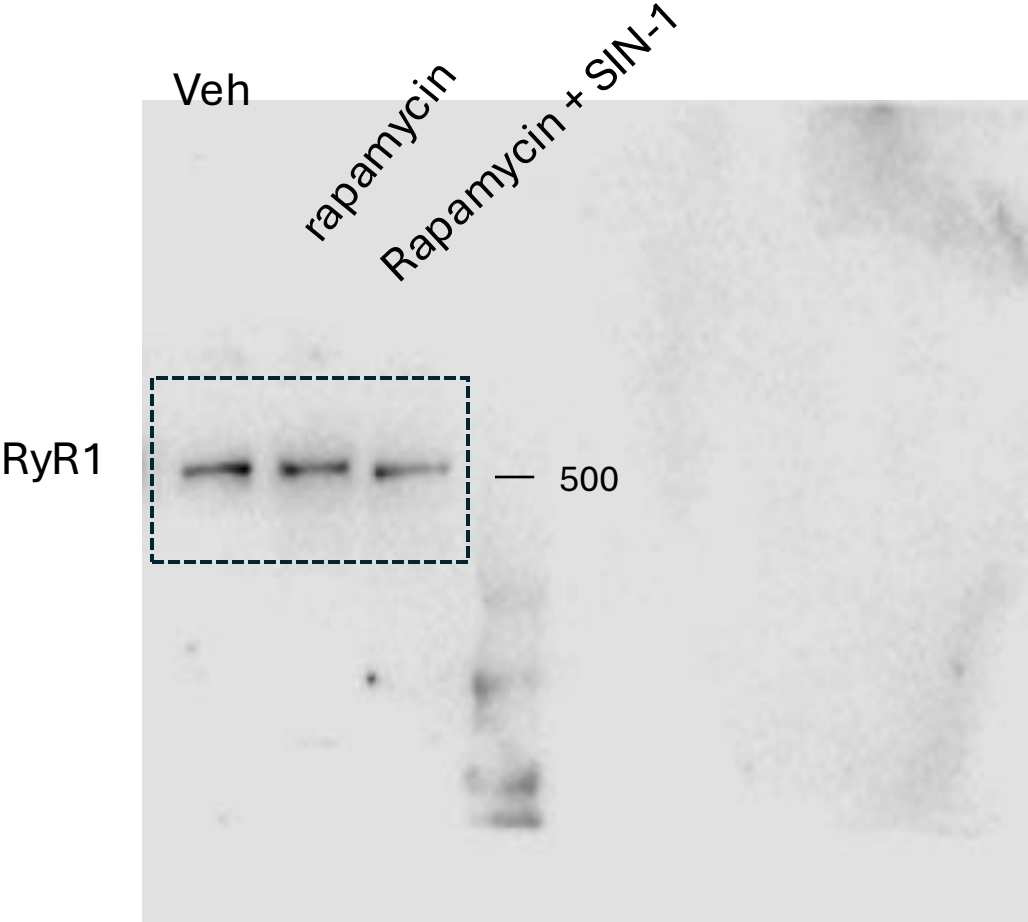

Supplement: SourceData F3 — is the source file for Fig. 3. [file JGP_202313515_SourceDataF3.pdf]
